# Inositol pyrophosphates activate the vacuolar transport chaperone complex in yeast by disrupting a homotypic SPX domain interaction

**DOI:** 10.1038/s41467-023-38315-w

**Published:** 2023-05-08

**Authors:** Joka Pipercevic, Bastian Kohl, Ruta Gerasimaite, Véronique Comte-Miserez, Sarah Hostachy, Thomas Müntener, Elia Agustoni, Henning Jacob Jessen, Dorothea Fiedler, Andreas Mayer, Sebastian Hiller

**Affiliations:** 1 Biozentrum, University of Basel, Spitalstrasse 41, 4056 Basel, Switzerland; 2 Department of Immunobiology, University of Lausanne, Chemin des Boveresses 155, CP51 1066 Epalinges, Switzerland; 3 Department of Chemical Biology, Leibniz-Forschungsinstitut für Molekulare Pharmakologie, Robert-Rössle-Straße 10, 13125 Berlin, Germany; 4 Institute of Organic Chemistry, University of Freiburg, Albertstraße 21, 79104 Freiburg, Germany; 5 Present Address: Max-Planck Institute for Biophysical Chemistry, Am Fassberg 11, 37077 Göttingen, Germany

**Keywords:** Solution-state NMR, Fungal physiology, Metabolic pathways, Proteins

## Abstract

Many proteins involved in eukaryotic phosphate homeostasis are regulated by SPX domains. In yeast, the vacuolar transporter chaperone (VTC) complex contains two such domains, but mechanistic details of its regulation are not well understood. Here, we show at the atomic level how inositol pyrophosphates interact with SPX domains of subunits Vtc2 and Vtc3 to control the activity of the VTC complex. Vtc2 inhibits the catalytically active VTC subunit Vtc4 by homotypic SPX–SPX interactions via the conserved helix α1 and the previously undescribed helix α7. Binding of inositol pyrophosphates to Vtc2 abrogates this interaction, thus activating the VTC complex. Accordingly, VTC activation is also achieved by site-specific point mutations that disrupt the SPX–SPX interface. Structural data suggest that ligand binding induces reorientation of helix α1 and exposes the modifiable helix α7, which might facilitate its post-translational modification in vivo. The variable composition of these regions within the SPX domain family might contribute to the diversified SPX functions in eukaryotic phosphate homeostasis.

## Abstract (web summary)

Pipercevic et al resolve how inositol molecules activate the VTC protein complex. The VTC complex stores phosphate in yeast and is controlled by SPX domains. The inositol molecules break an interaction between SPX domains to activate the complex.

## Introduction

Inorganic phosphate (P_i_) is an essential building block for biomolecules and therefore a crucial nutrient and metabolite. Phosphate homeostasis is tightly controlled in all living organisms. In yeast, the proteins involved in phosphate homeostasis include phosphate importers, putative phosphate exporters, phosphate scavenging proteins, P_i_-responsive transcription regulators, and vacuolar transport proteins^1^. Most of these proteins contain the SPX (Syg1/Pho81/Xpr1)-domain^2,3^. SPX domains regulate P_i_ transport and activate P_i_ storage via the VTC complex^4^. In humans, the SPX domain of Xpr1 facilitates P_i_ export^5–7^. In plants, the SPX domain of PHO1 enables P_i_ export^8^ and the SPX domain of stand-alone SPX proteins inhibits P_i_-starvation response (PSR) transcription factors under P_i_ sufficient conditions^9–13^. These functions are modulated by inositol phosphates and pyrophosphates (IP_x_) such as 1,5-bis-diphospho-inositol tetrakisphosphate (1,5-IP_8_)^14^, and 5-diphospho-inositol tetrakisphosphate (5-IP_7_)^15,16^, which bind directly to SPX domains^8^.

So far, six structures of the α-helical protein domain SPX have been determined at the atomic level^8,17^. The SPX domain is comprised of six helices α1–α6 and contains a binding pocket for IP_x_, localized between helices α1, α2, and α4^8^. Mutations in this binding pocket typically impair the respective protein function^6,8,15,16,18^. Mutations outside the binding pocket^5,8,19–22^ and in the linker connecting the SPX to an adjacent domain^5,23^ have also been shown to affect the function. For the SPX domain of human Xpr-1, several mutations have been identified which lead to primary familial brain calcification (PFBC) manifested in neuropsychiatric abnormalities and movement disorders^5,24^. For plants, mutations in SPX domains lead to malfunctions like hyperaccumulation of phosphate, resulting in growth defects^8,13^.

Up to now, only the interactions of SPX proteins from *Arabidopsis thaliana* and rice have been studied in detail. The SPX proteins AtSPX1 and OsSPX4 interact with the P_i_ starvation response transcription factors (PHRs). Hereby, inositol pyrophosphates act synergistically to the interaction between the SPX protein and a coiled-coil domain in PHR^25–28^. In vitro, SPX binds to the coiled-coil domain in the low micromolar (μM) range in the presence of 1,5-IP_8_ and 5-IP_7_ and ten times less efficient in the presence of inositol phosphate, IP_6_^8,13^.

In yeast, the VTC complex plays a crucial role in phosphate homeostasis^29^. Upon activation, it synthesizes polyphosphate chains from cellular ATP, coupled with the translocation of these polyphosphate chains into the vacuole lumen^30–32^. The VTC complex exists in two isoforms, each comprised of three core proteins: Vtc1/2/4 or Vtc1/3/4 with stoichiometry 3:1:1. Vtc1 and Vtc4 are thus obligate components, while Vtc2 and Vtc3 act as mutually exclusive isoforms. Vtc2 and Vtc3 have similar topology and many critical residues are conserved. Vtc5 can associate with either isoform of the VTC complex to activate it^31^. Only Vtc4 harbors ATPase activity required for polyphosphate generation, while the transmembrane parts of all subunits together form a channel comprised of 15 transmembrane helices^32–34^. Under phosphate-rich conditions, the vacuolar VTC complexes are mostly the Vtc3-containing isoform, while at the cellular periphery, most VTC complexes are the Vtc2-containing isoform. The latter gets translocated to the vacuole under phosphate starvation conditions. In vitro studies have shown that inositol phosphates and pyrophosphates activate polyphosphate generation by the yeast VTC complex^8,16^. Subunits Vtc2–5 are comprised of a cytosolic N-terminal SPX domain and a central triphosphate tunnel metalloenzyme (TTM) domain as well as an uncharacterized C-terminal membrane domain. The small protein Vtc1 consists of solely this uncharacterized membrane domain^35–37^.

The VTC complex as a whole has been characterized at the structural level by cryo-electron microscopy^33,34^, while structures of the individual TTM domains of Vtc2 and the SPX and TTM domains of Vtc4 have been solved by X-ray crystallography^8^. In the structure of the VTC complex, the TTM of Vtc4 is localized on top of the channel and is surrounded by the SPX domains of Vtc3 and Vtc4, as well as the TTM domain of Vtc3. In one of the structures, the TTM domains are held together by an IP_6_ molecule, which is, however, not influencing polyphosphate activity^33^. It is suggested that the positioning of TTM4 domain on top of the channel enables to couple synthesis of polyphosphate to its membrane translocation^31,33,34^. Polyphosphate synthesis assays revealed that different IP_x_ molecules have different potency to stimulate polyphosphate synthesis. The cellular IP_7_ regioisomers 1-IP_7_ and 5-IP_7_ have EC_50_ values between 350 nM and 500 nM, whereas 1,5-IP_8_ has an EC_50_ of 15 nM corresponding to a 20-fold higher potency^16^. Though IP_6_ binds the SPX domains of VTC, it is not a significant activator of the complex, yielding a 10-fold lower apparent maximal activity than 1,5-IP_8_, and only above a concentration of 100 μM^8,16^, which is at the upper limit or above the physiological levels (10–100 μM) observed in cells from various organisms^38–42^. 1,5-IP_8_ was suggested to be relevant for VTC activity in vivo^16,43^. Research of the past decade revealed that mutations in the IP_x_ binding pocket impair the function of the VTC complex^8,16^.

The molecular mechanism of SPX domains as regulators of polyphosphate synthesis has remained unclear. Here, we characterized the SPX domain of Vtc2 and its interaction with the SPX and TTM domains of Vtc4, and with IP_x_ molecules, employing solution NMR spectroscopy, microscale thermophoresis (MST), nano differential scanning fluorimetry (nanoDSF) and VTC activity assays. We find that the SPX domains of Vtc2 and Vtc3 inhibit VTC activity via SPX–SPX interactions with Vtc4 in an IP_x_-dependent manner.

## Results & discussion

### Characterization of SPX2 in aqueous solution

**Fig. 1:**
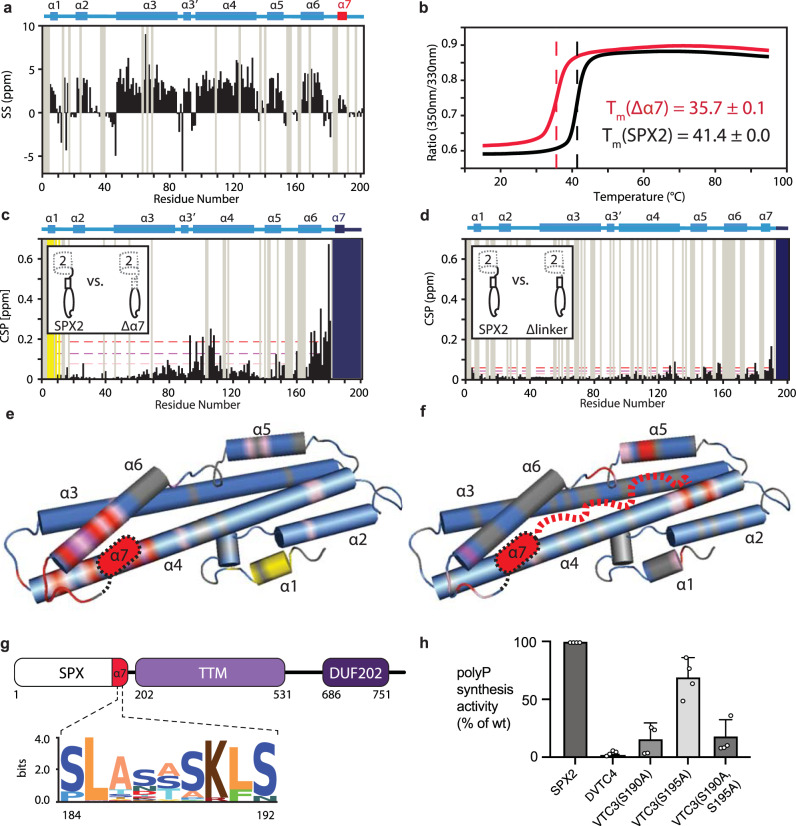
SPX2 contains a functionally relevant helix α7. **a** Identification of secondary structure elements in SPX2 by secondary chemical shifts (SS). **b** Thermal stability of SPX2 variants determined by nanoDSF. **c**, **d** Chemical shift differences of SPX2 upon truncation of helix α7 or the linker region. Yellow – residues experiencing intermediate chemical exchange, gray – residues not assigned, dark blue – residues that were deleted. NMR buffer was used for all experiments. **e**, **f** Chemical shift differences observed in C and D, plotted on a structural model of SPX2. Yellow – residues experiencing intermediate chemical exchange, gray – residues not assigned, pink/magenta/red – low/medium/high chemical shift differences (μ + 0.2 σ/μ + 0.8 σ/μ + 1.5 σ), where μ = average and σ = standard deviation. **g** Sequence motif of helix α7 identified by alignment of 69 Vtc2 homologues, shown with the residue numbering of SPX2. **h** Polyphosphate synthesis by purified vacuoles carrying Vtc1/3/4/5 or mutants thereof. Graphs show the means and SEM; *n* = 3 replicates. All substituted proteins were expressed at similar levels (95–110%) as the wild-type proteins. Source data are provided as a Source Data file.

For the characterization of the SPX domain of Vtc2 (SPX2) in aqueous solution by NMR spectroscopy, near-complete sequence-specific resonance assignments of the protein backbone were obtained by standard triple-resonance experiments (Supplementary Fig. 1a). Secondary chemical shifts derived from ^13^C_α_ and ^13^C_β_ nuclei display the presence of secondary structure elements in the protein. The data show that in an aqueous solution, SPX2 comprises a total of eight α-helices (Fig. 1a). Seven of these helices, α1–α6, and the short helix α3’ have been previously resolved in crystallographic structures of homologous SPX domains^8^. In addition, our data identify the short helix α7 at the C-terminus of SPX2, located at positions 184–192. This segment has a helical propensity of around 50%, as evidenced by the secondary chemical shift values, suggesting that it populates a rapidly interconverting ensemble of helical and random-coil conformations. This finding is underlined by reduced signal intensities for these residues (Supplementary Fig. 1b) and NMR relaxation measurements of the protein backbone dynamics, which show that helix α7 features increased dynamics on the ps–ns timescale compared to the other helices (Supplementary Fig. 2a–c). Furthermore, Alphafold predicts a helical segment for residues 185–188 in Vtc2 and the corresponding region in Vtc3 (Supplementary Fig. 2d)^44,45^.

Next, we performed nanoDSF measurements to quantify the contributions of helix α7 to the stability of the SPX domain. The presence of this helix substantially stabilizes the SPX domain, as evidenced by an increase of the domain melting temperature by 5.7 °C in the presence of the helix, identifying this region as an integral structural part of the domain (Fig. 1b). By analyzing the chemical shift perturbations caused by truncation of helix α7, we localize α7 between helices α4 and α6 (Fig. 1c, e). In addition, we conclude that the unstructured C-terminal part of the domain (residues 193–201) can dynamically reach all the way to the IP_x_ binding pocket located at helices α1 and α4 (Fig. 1d, f)^8^. This interpretation is in agreement with H/D exchange NMR data showing that two regions in SPX2 are exchange-protected – one region near the known binding pocket and a second region between helices α4 and α6 on the opposite side of the molecule (Supplementary Fig. 2e, f).

Helix α7 likely has a functional role in VTC and presumably in a few additional SPX-containing proteins, which we conclude based on the following points. Firstly, helix α7 of Vtc2 and Vtc3 in *S. cerevisiae* contains several reported phosphorylation sites. Secondly, several SPX proteins in *S. cerevisiae* and *A. thaliana* contain phosphorylation sites in the region adjacent to helix α7 (Supplementary Table 1). Thirdly, the human mutation L218S, which causes PFBC disease, maps in the region adjacent to helix α7 (Supplementary Table 1). Fourthly, the amino acid sequence motif SLASASKLS (Fig. 1g), albeit generally rare, is found in a few SPX-containing proteins other than VTC 2/3 (Supplementary Table 2). Thereby, the variability of α7 suggests it to be a diversification module of SPX domains to facilitate distinct functions in P_i_ homeostasis. To test the hypothesis of helix α7 having a functional role in VTC, we probed it by a single point mutation. An in vitro activity assay is available for this complex, which monitors polyP synthesis by isolated vacuoles^31^. Indeed, substituting Ser190 of the predicted helix α7 in Vtc3 (^187^SLA**S**TSKLS^195^) that is homologous to Ser187 in Vtc2 (^184^PLA**S**ASKFS^192^) reduced the apparent activity of polyP synthesis by 75% (Fig. 1h).

### The functional role of the SPX domains in the VTC complex

**Fig. 2:**
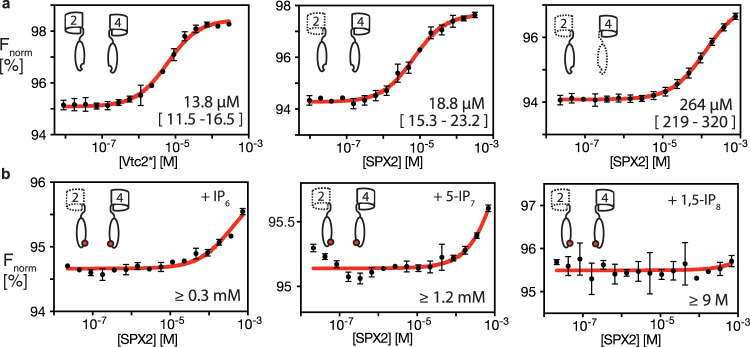
Vtc2 and Vtc4 interact via their SPX domains in an IP_x_-dependent manner. **a** Binding affinities of Vtc2* to Vtc4*, SPX2 to Vtc4*, and SPX2 to TTM4, as determined by microscale thermophoresis (MST). **b** Binding affinities of SPX2 to Vtc4* in the presence of 2.5 mM IP_6_, 5-IP_7_, or 1,5-IP_8_ determined by MST. Cartoons illustrate the constructs used in each experiment. The resulting dissociation constants are given along with a 95% confidence interval or as a lower limit. Graphs show the means and SEM; *n* = 3 replicates. Source data are provided as a Source Data file.

The vacuolar VTC complex is comprised of SPX-containing proteins and contributes to yeast P_i_ homeostasis while it is synthesizing and storing imported phosphates in the form of polyphosphates^46^. While the role of the Vtc4 subunit of the VTC complex is to catalyze polyphosphate chains by ATP hydrolysis via its central TTM domain, the transmembrane subunit Vtc1 enables polyphosphate transport and the SPX domain-containing subunit Vtc2/Vtc3 are likely to perform regulatory functions^32,33^. We investigated the role of the soluble domains of Vtc2 and Vtc4 using protein constructs that comprised the respective SPX and TTM domains but lacked the transmembrane domains. We annotate these constructs as Vtc2* and Vtc4*, respectively. MST measurements showed that Vtc2* and Vtc4* interact directly, with a dissociation constant *K*_D_ = 13.8 µM (11.5–16.5) (Fig. 2a). In the cellular context this interaction can be readily expected to be substantially stronger, because both domains are located on the cytosolic face of the vacuolar membrane, where they are held at effectively increased local concentration by their joint integration in the VTC complex. To localize the binding interface, we used single-domain constructs of the SPX or TTM domains of Vtc2 and Vtc4. The SPX domain of Vtc2 alone (SPX2) bound to Vtc4* with similar affinity as Vtc2*, indicating that it harbors the binding site and that the TTM domain of Vtc2 (TTM2) does not significantly contribute to the interaction (Fig. 2a). Then, the TTM4 domain alone bound SPX2 with a ~ 20-fold reduced affinity compared to Vtc4* (Fig. 2a), suggesting that the interaction is mediated by the SPX4 domain. As a control, we found that TTM2 did not show detectable binding to Vtc4* (Supplementary Fig. 3a). Probing the interaction between SPX2 and SPX4 directly was experimentally not possible due to limited biochemical stability of purified SPX4 domain in the absence of the TTM4 domain. We therefore probed the existence of this interaction by co-immunadsorption (co-IA). The SPX domain of Vtc4 was expressed in yeast and incubated on beads with recombinant SPX2. Co-IA showed a clear signal for SPX2, demonstrating the presence of the SPX2–SPX4 interaction in native-like conditions (Supplementary Fig. 3b). This signal was not observed when the beads were incubated with 1,5-IP_8_. Taken together, these data show that an interaction between Vtc2* and Vtc4* exists and is mediated by the SPX domains of the two proteins.

The SPX2–Vtc4* interaction comprises substantial electrostatic contributions, as suggested by the reduction of binding affinity upon an increase of the salt concentration (Supplementary Fig. 3c). A direct titration of SPX2 with sodium chloride monitored by NMR revealed that salt-sensitive residues are located mostly in helices α1 and α7, as evidenced by substantial line broadening of α1 residues and large chemical shift perturbations in α7 (Supplementary Fig. 3d), suggesting that these parts of the protein are specifically involved in the interaction. Indeed, truncation of either helix α1 or helix α7 reduced the SPX2 binding affinity to Vtc4* dramatically by ~35–70-fold (Supplementary Fig. 3e). In addition, either of the single point mutations of serine 187 or 189 to alanine in helix α7 reduced the binding affinity between SPX2 and Vtc4* by factors 2–5 (Supplementary Fig. 3f), suggesting these residues to be part of the interaction interface. Altogether, the data show that SPX2 binds to Vtc4* via an interface located among helix α1 or helix α7.

### Regulation of the SPX2–SPX4 interaction by IP_x_

Next, we investigated the effect of different inositol phosphates on the SPX2–Vtc4* interaction, because these ligands stimulate VTC polyphosphate generation^8,16^. Our MST measurements revealed that either IP_6_, 5-IP_7_, or 1,5-IP_8_^47^, (summarized as IP_x_), disrupted the SPX2–Vtc4* interaction (Fig. 2b, Supplementary Fig. 4a). Among the three ligands, the highly phosphorylated IP_8_, which is the most potent stimulator of VTC^16^ had the most pronounced effect, by reducing the affinity between SPX2 to Vtc4* by more than five orders of magnitude. 5-IP_7_ decreased the affinity 85-fold and IP_6_ by more than 20-fold. The findings were corroborated by solution NMR spectroscopy and cross-linking experiments (Supplementary Fig. 4b, c). SPX2 in the presence of Vtc4* features strong NMR line broadening, leading to an essentially empty 2D [^15^N,^1^H]-NMR spectrum. When IP_6_ was added to the sample, narrow resonance lines were restored and the characteristic spectrum of apo SPX2 was observed (Supplementary Fig. 4b). This observation is indicative of an VTC4*–SPX2 interaction that quenches the SPX2 resonances either due to its high molecular weight, or by the presence of multiple binding modes leading to conformational heterogeneity and dynamic line broadening. Furthermore, intermolecular chemical cross links between SPX2 and Vtc4* that formed in the absence of IP_6_ were significantly diminished in the presence of IP_6_ (Supplementary Fig. 4c). Together, these experiments show that SPX2 binds to Vtc4* and that this interaction is disturbed by IP_x._

**Fig. 3:**
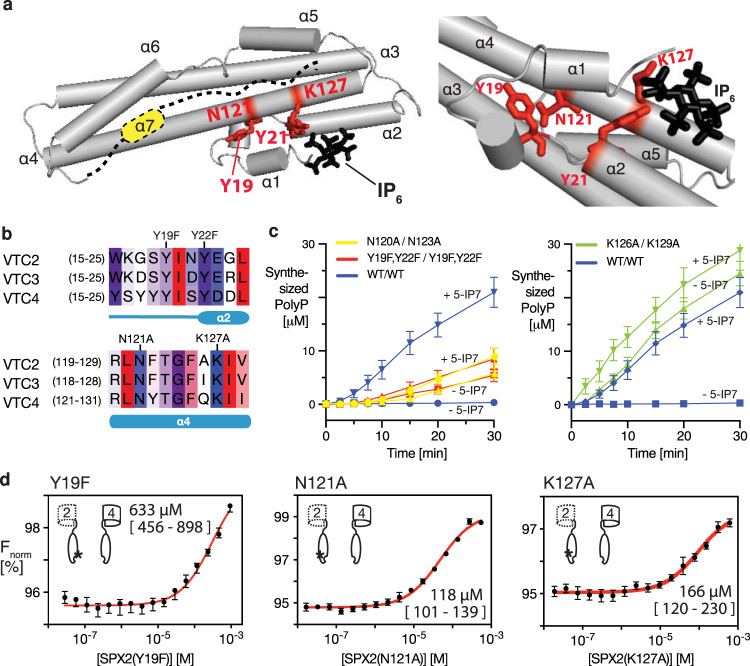
Specific mutations in SPX2 disrupt binding to Vtc4* and cause constitutive activity of the VTC complex. **a** Structural model of SPX2 with the positions of four conserved amino acids labeled in red. The expected position of bound IP_6_ ligands is shown in black. **b** Multiple sequence alignment of SPX-containing Vtc proteins, colored according to consensus hydrophobicity (high–red; low–blue)^76^. The position of activating mutations is indicated on top. **c** Polyphosphate synthesis by purified vacuoles carrying Vtc1/3/4/5 in the absence and the presence of 1 µM 5-IP_7_. 5 µg/ml of purified vacuoles were incubated with an ATP regenerating system to allow in vitro synthesis of polyP by VTC. At the indicated time points, the reaction was quenched with EDTA and detergent, DAPI was added and polyP was quantified by fluorescence of the resulting DAPI/polyP complexes. Graphs show the means and SEM; *n* = 3 replicates. All substituted proteins were expressed at similar levels (95–110%) as the wildtype proteins. **d** Binding affinities of pseudo-active mutations (Y19F, N121A, K127A) in SPX2 to Vtc4* determined by MST. Graphs show the means and SEM; *n* = 3 replicates. The resulting dissociation constant is indicated, along with a 95% confidence interval. Asterisks display the mutations in the cartoon scheme. Source data are provided as a Source Data file.

In the context of the VTC complex, IP_x_ thus likely activates VTC activity by disrupting the inhibitory interaction between the Vtc2/Vtc3 and Vtc4 subunit. We tested this assumption using substitutions that destabilize this interaction. Functional yeast VTC complexes in vacuoles can be isolated only for the Vtc3-containing isoform and not the Vtc2-contining isoform. The functional effect of destabilizing mutations was therefore tested using the Vtc3-containing VTC complex isoform, whose activity can be assayed biochemically in the isolated organelle^31,37^, under the assumption that the conclusions established on isoform Vtc2 are transferrable to isoform Vtc3. K127 belongs to the lysine surface cluster constituting the binding pocket of IP_x_, while residues Y19, Y22, and N121 reside in the proximity of the binding pocket in the X-ray structures of SPX domain in Vtc4^8,17^. We simultaneously introduced substitutions into VTC3 and VTC4, affecting conserved residues corresponding to SPX2 Y19 and Y22 (*vtc3*^Y19F,Y22F^/*vtc4*^Y19F,Y22F^), N121 (*vtc3*^N120A^/*vtc4*^N123A^), or K127 (*vtc3*^K126A^/*vtc4*^K129A^) (Fig. 3a, b). In the absence of ligand, the wildtype VTC complex showed only marginal activity, while the *vtc3*^N120A^/*vtc4*^N123A^-containing VTC complex was partially activated (Fig. 3c). Then, upon addition of 1 µM 5-IP_7_, the wildtype complex was activated 50-fold, while the *vtc3*^N120A^/*vtc4*^N123A^-containing VTC complex was hardly further stimulated. A similar ligand-independent partial activation with simultaneous insensitivity to ligand was observed for the *vtc3*^Y19F,Y22F^/*vtc4*^Y19F,Y22F^ complex. In contrast, *vtc3*^K126A^/*vtc4*^K129A^ showed a very high activity already in the absence of ligand that corresponded to the maximal activity that could be observed with the wildtype complex stimulated by 5-IP_7_ and this high basal activity also increased only marginally upon the addition of ligand (Fig. 3c). We then introduced these mutations into the SPX2 domain in order to characterize their effect on the in vitro binding to Vtc4*. Strikingly, these single mutations weakened binding to Vtc4* by factors of 8–45 compared to wildtype (Fig. 3d). Together, the data thus show that the SPX2/3–SPX4 interaction can be disrupted by either IP_x_ binding or by specific point mutations to activate the VTC complex. The interaction of the SPX domain Vtc4 with that of Vtc2/Vtc3 thus appears to have an inhibitory effect on the VTC complex. Notably, this does not exclude that IP_X_-bound SPX, once dissociated from the SPX–SPX dimer stimulates VTC.

### Structural insights

**Fig. 4:**
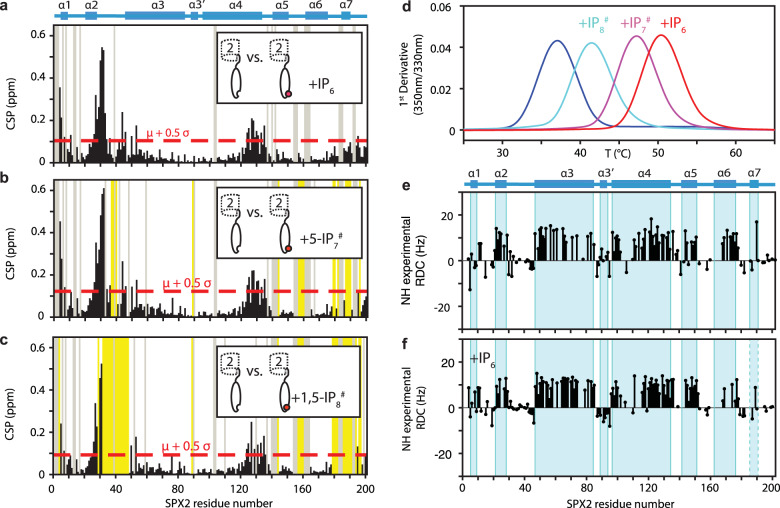
IP_x_ binding perturbs structure and dynamics of the SPX2 domain to variable degree. **a**–**c** Chemical shift perturbation plots of SPX2 apo- vs. holo-states with 10-fold molar excess the non-hydrolysable ligands IP_6_, 5-IP_7_ and 1,5-IP_8_, respectively, in NMR buffer. Yellow – residues experiencing intermediate chemical exchange, gray – residues not assigned. **d** Thermal stability of SPX2 in the presence of different ligands determined by nanoDSF. **e**, **f** Residual dipolar couplings (RDCs) of SPX α-helices in the absence and presence of 10-fold molar excess of IP_6_. Source data are provided as a Source Data file.

Next, we wanted to explore the molecular mechanism of IP_X_ binding to the SPX2 domain on the structural level by solution NMR experiments. We titrated SPX2 with the phosphoinositol IP_6_ or with the stable, non-hydrolysable 5-methylene-bisphosphonate inositol pentakisphosphate (5-IP_7_^#^) or 1,5-bisdiphosphoinositol tetrakisphosphate (1,5-IP_8_^#^) (Supplementary Fig. 5). Binding of either ligand caused large chemical shift perturbations in the helices α1 and α2, and in the C-terminal part of α4 (Fig. 4a–c), confirming the known binding pocket of inositol polyphosphates^8^. Moreover, large chemical shift perturbations were observed for the N-terminal part of helix α3 and its adjacent loop α2–α3, as well as for the loop adjacent to helix α7. Thereby, residues K30, E31 and D32 of SPX2 undergo the largest chemical shift perturbations upon IP_6_ binding (Fig. 4a–c), indicating a strong conformational change either due to direct interaction with the ligand or due to an allosteric mechanism. In line with these findings, in two crystal structures of other SPX domains, human Xpr1 (PDB 5IJH) and Gde1 (PDB 5IJJ), K30 interacts with one of the phosphates of IP_6_^8^.

Notably, the interactions of SPX2 with the three IP_X_ molecules showed clear differences in their conformational dynamics. For the interaction with IP_6_, fast chemical exchange was observed, whereas 5-IP_7_^#^ and 1,5-IP_8_^#^ showed intermediate exchange for the helix α7 region and the loop regions bridging helices α2/α3, α5/α6 and α6/α7 (Fig. 4a–c), with 1,5-IP_8_^#^ having a stronger effect than 5-IP_7_^#^. Such line broadening can occur due to conformational exchange between the apo- and the holo-state, including local conformational heterogeneity of the holo-state. Measurements of protein stability by thermal denaturation in the presence of these ligands showed that the IP_x_ molecules stabilized the SPX domain to different degrees, which matched the observed dynamics. IP_6_ thermally stabilized SPX2 the most, by 13 °C, followed by 10 °C through 5-IP_7_^#^ and 4 °C by 1,5-IP_8_^#^ (Fig. 4d and Supplementary Table 3). Considering that all ligands bind with similar binding affinities (Supplementary Fig. 6a), these data suggest a differential conformational plasticity of three resulting protein–ligand complexes: IP_6_ forms a stable and compact protein-ligand complex with SPX2, while 5-IP_7_^#^ and 1,5-IP_8_^#^ engage in a dynamic interaction mode characterized by high conformational entropy.

Next, we inspected the effect of three of the four VTC-activating point mutations on SPX2 at the structural level. The chemical shifts of the backbone resonances were compared between the single mutants and the wild-type. Strikingly, the chemical shift changes caused by the mutations were found to be localized in helices α1, α2, and α4, which comprise the IP_x_ binding pocket, and additional changes were observed in helix α3 and loop α5-α6 (Supplementary Fig. 6b–d). Some variations were observed between the individual single-point mutants. Mutants Y19F and N121A caused perturbations in loops α1-α2, α2-α3, and helix α5, but these were not observed in the K127A mutant. These findings can be well rationalized, because the residues homologous to Y19F and N121A interact with each other in available SPX structures and K127A is more distant^8^. The VTC-activating mutations thus have a similar effect on the SPX2 structure as IP_x_ binding, in agreement with a shared functional role. Presumably, the mutations stabilize a state that corresponds to the holo-form of SPX2 and destabilizes the apo form. Since the apo form interacts with Vtc4, that interaction is weakened and the complex activated.

Furthermore, we probed the extent and relative orientation of the α-helices of the SPX2 domain upon ligand binding by residual dipolar couplings (RDCs). The data showed that the binding of IP_6_ to SPX2 did not lead to changes of secondary structure elements in helices α1−α6 (Supplementary Fig. 6e–f). This observation agrees well with the available crystal structures of apo- and holo SPX4 (PDB: 5IIG, 5IIQ, and 5IJP)^8^. For α7, the secondary chemical shifts were not detectable in the holo-form and it thus remains unclear whether the helix forms in the holo form. An analysis of the relative orientations of the helices using residual dipolar couplings (RDCs) showed that the global alignment tensor of the protein was aligned to the prolate ellipsoid shape of SPX2 (Supplementary Fig. 7). Upon binding of IP_6_, the tensor component along the long axis was essentially maintained, while changes occurred in the plane transverse to it. This finding is in full agreement with structural changes around the ligand binding site but no major changes in the elongated shape of the protein. The orientation of the individual helices α2−α6 was maintained (Fig. 4e–f, Supplementary Fig. 7). In contrast, the orientation of helix α1, the adjacent loop, and helix α3’ changed upon the addition of IP_6_, such that helix α1 was aligned in the same relative orientation as the other helices in the holo-form.

In summary, the IP_x_-bound states and the pseudo-activating mutations represent the active state of SPX2. Ligand binding does not alter the secondary structure elements of α1−α6, but induces a reorientation of helix α1 with its adjacent loop and helix α3’. The helix α7 experiences a change in secondary structure from α-helix into a random-coil. The SPX domain thus experiences ligand-dependent conformational plasticity.

### A functional model for VTC activity control

**Fig. 5:**
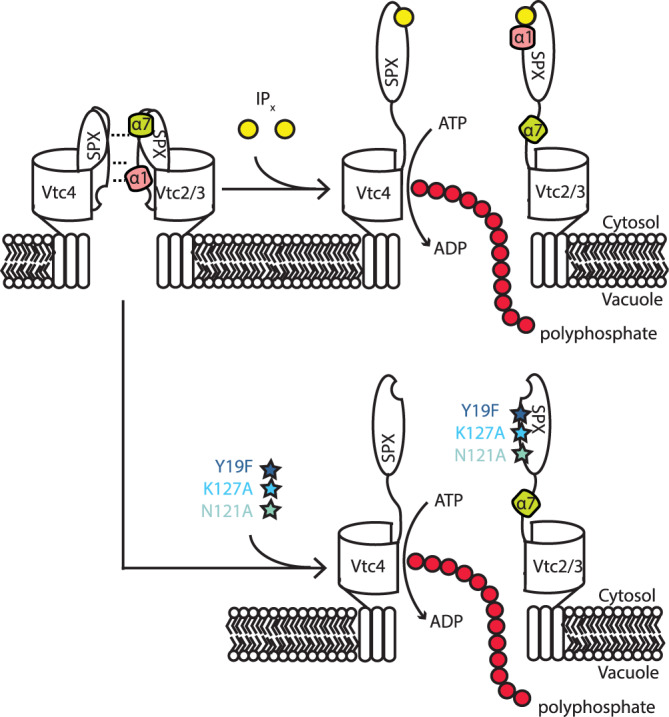
Mechanistic model of regulation in the VTC complex. An SPX–SPX interaction of Vtc4 and Vtc2/3 is based on electrostatic interactions of helix α1 and helix α7. IP_x_ or pseudo-active mutations lead to a ligand-induced helix α1 reorientation, liberate helix α7 and hence disrupt the interaction between Vtc4 and Vtc2/3. This disruption initiates an activation of VTC.

Taken together, our structural, biophysical, and activity data lead us to propose a model for the regulation of the VTC complex. We hypothesize that in the inactive state, a homotypic interaction between the SPX domain of Vtc4 and the SPX domain of Vtc2 or Vtc3 prevents activation of the VTC complex (Fig. 5). In the currently available EM structures of the VTC complex^33,34^, these SPX domains do not contact each other. Therefore, these structures might represent conformations that the SPX domains attain once liberated from the IP_x_-free, SPX–SPX dimerized state. This can only be judged once the relevance of the contact interfaces will have been systematically tested by mutagenesis and further biophysical analysis. Our model of SPX–SPX dimerization as a means to inactivate VTC is, however, in agreement with the observation that the SPX domain of Vtc4 must be available in order to activate VTC.

Starting from the inactivated state, the binding of IP_x_ signaling molecules as well as the activating mutations weaken the SPX–SPX interaction, thus activating the VTC complex. Specifically, the binding of inositol phosphates and pyrophosphates to the SPX ligand binding pocket located at helices α1, α2, and α4 leads to a reorientation of helix α1 and its adjacent loop, which is likely responsible for the release of the VTC inhibition. The conformational dynamics upon ligand binding establishes the ligand order 1,5-IP_8_ > 5-IP_7_ > IP_6_ for the stability of the protein–ligand complex, reducing dynamics, and increasing liberation of Vtc4*. A corresponding hierarchy of agonist potency was revealed by in vitro experiments on VTC activity, where 1,5-IP_8_ was the strongest activator^8,16^. Interestingly, 1,5-IP_8_ levels undergo large Pi-induced changes in yeast, human cells, and in plants^43,48,49^. Helix α7 appears to have an important functional role, as it participates in the interaction of Vtc components, exhibits ligand-induced conformational changes, and influences VTC activity. Since phosphorylation sites were found in the serine/threonine-rich region around helix α7 (Supplementary Table 1), it is tempting to speculate that these residues could become exposed to post-translational modification or protected in a ligand-dependent manner, allowing further regulation of the VTC complex. Proteome-wide analyses have indeed identified multiple phosphorylations in helix α7 of Vtc2/Vtc3^50–55^. Remarkably, the region including helix α7 of SPX3 is disordered in the recently obtained structures of VTC complex^33,34^, in full agreement with our model that expects this helix to interact with other parts of the complex in the inactivated but not the activated form of the VTC complex.

While there is no IP_6_ molecule bound to SPX3 in the published structures of the VTC complex^33,34^, a comparison of the location of helix α1 to other holo-SPX structures suggests that SPX3 is in a holo-like state. IP_6_ might have been bound but not fixed tight enough, or it might be in a conformational ensemble state in the binding pocket, preventing its detection by cryo-EM. In the structure of IP_6_-bound VTC, the SPX3 and SPX4 domains do not interact directly with each other. This is in agreement with our model, which suggests that the disruption of the SPX3–SPX4 interaction by inositol pyrophosphates leads to the rearrangement of domains and activation of VTC. Our findings thus make predictions for a yet unsolved inactive form of VTC, in which SPX domains interact in a way to inhibit complex activity.

Overall, the data presented in this work suggest that oligomerization of its SPX domains may inhibit the VTC complex, and that IP_x_ ligands relieve this inhibition by dissociating the domains. The IPx binding pocket of other SPX domains and its adjacent regions play a crucial role also for the inhibition or activation of other SPX-carrying proteins^6,8,15,18^. Because the ligand binding pocket is highly conserved in SPX-domains, but the region around helix α7 is variable, we could imagine that the latter contributes to the diversification of function among the SPX-domain family. The disruptive effect of IP_x_ on SPX oligomerization shown here could possibly enable the formation of additional molecular contacts of the SPX domains with the target proteins, which may further contribute to their regulation.

## Methods

### Protein expression and purification

Starting from Vtc2(1–182) with C-terminal His_5_-tag in pET vector (pMH-HC)^8^, a TEV cleavage site was engineered adjacent to the His_5_-Tag (SPX2∆α7). A stretch of residues was inserted by Q5 Kit (*New England Biolabs*) to generate longer Vtc2 constructs: Vtc2(1–193), SPX2∆linker, and Vtc2(1–201), SPX2. The site-specific mutations were introduced into SPX2 by the QuikChange II mutagenesis protocol (*Stratagene*) by using Phusion DNA polymerase (*Thermo Scientific*) and were verified by Sanger sequencing. SPX2 proteins were recombinantly expressed in *E. coli* Lemo21(DE3) BL21 cells (*New England Biolabs*). Cells were grown in LB medium for expressing unlabeled proteins or M9 minimal medium supplemented by ^15^NH_4_Cl for [*U*-^15^N]-labeled proteins. For expression of [*U*-^2^H,^15^N] or [*U*-^2^H,^15^N,^13^C]-labeled constructs, M9 minimal medium supplemented by either [*U*-^2^H]- or [*U*-^2^H,^13^C]-glucose, respectively, as well as ^15^NH_4_Cl and D_2_O was used. All cultures were supplemented with 30 mg kanamycin per 1 L medium. Cells were grown at 37 °C to an optical density (600 nm) of 0.4–0.6. The temperature was reduced to 16 °C and after 1 h, expression was induced with 0.3 mM isopropyl-β-thiogalactopyranoside (IPTG). Cells were harvested after 12 h and resuspended in 50 mL of buffer A (20 mM Tris-HCl pH 8.0, 500 mM NaCl, 2 mM β-mercaptoethanol, 20 mM imidazole, 0.5% Triton, 2 mM MgCl_2_) and 500 units of Turbo Nuclease (*BioVision*). The cells were disrupted with lysozyme and a microfluidizer and the cell lysate was centrifuged at 42’500 g for 40 min at 4 °C. The cleared lysate was filtered via 0.22 μm membrane and afterwards the protein was isolated by affinity chromatography protocol. The lysate was loaded onto Ni-NTA based HisTrap 5 ml (*GE Healthcare*) column equilibrated with buffer A. The protein was eluted with a linear gradient 0–60 % by twenty column volumes (CV) of buffer A supplemented with 0.5 M imidazole. The fractions containing the protein were collected and TEV protease was added. The sample was incubated at 4 °C overnight. After buffer exchange against buffer A, the cleaved His-tag was removed by a reversed His-trap purification step. The protein contained in the flow through fractions was collected and diluted 10-fold in buffer B (50 mM Na-Ac pH 4.5, 20 mM NaCl) and subsequent purified by cation exchange chromatography. The sample was applied on equilibrated HiTrap SP HP (*GE Healthcare*). The protein was eluted with a 0–100% linear gradient of buffer B supplemented by 1 M NaCl. The pure protein sample was subjected to Superdex 75 pg 16/600 (*GE Healthcare*) for buffer exchange to NMR buffer (25 mM HEPES pH 7.0, 250 mM NaCl, 0.5 mM EDTA and 0.5 mM TCEP). The central fractions of the elution peak were collected and used for all subsequent experiments. The cation exchange chromatography step was skipped for SPX2∆α1 due to protein instability caused by the pH jump and it was slightly modified for SPX2∆α7, such that a different buffer B was used (20 mM MES, pH 6.0 and 20 mM NaCl).

pET27b(+)-based Vtc2(1–553), Vtc2*, and Vtc4(1–487), Vtc*, as well as TTM4 of Vtc4(192–487) with a C-terminal TEV cleaving site and a His_10_-tag were ordered from *GenScript*. These proteins were expressed and purified like the SPX2 proteins with following modifications. Affinity chromatography by HisTrap contained several wash steps in a length of two CV. The protein was first washed with buffer A, followed by a wash with buffer A supplemented with 200 mM potassium phosphate and wash with buffer A containing 1 M NaCl in total with a subsequent washing by buffer A. Finally, linear gradient (0–100 %) of buffer A supplemented with 0.5 M imidazole (20 CV) eluted the protein. A cation exchange chromatography by SP HP (*GE Healthcare*) was not performed and the size-exclusion chromatography was conducted by Superdex 200 pg 16/600 (*GE Healthcare*) in the NMR buffer.

### NMR spectroscopy

NMR experiments were recorded at 27 °C on 600 and 900 MHz NMR spectrometers (*Bruker*) with cryogenic triple resonances probes. Unless stated otherwise, the NMR buffer contained 25 mM HEPES pH 7.0, 250 mM NaCl, 0.5 mM EDTA, 0.5 mM TCEP and 5% D_2_O. Inositol phosphate was purchased from *Calbiotech* while stable non-hydrolysable analogs of inositol pyrophosphate were synthesized as described^56–58^.

### Backbone experiments

The assignments on backbone amides were obtained on 500 μM [*U*-^2^H,^15^N,^13^C]-labeled SPX2 in the absence and presence of 10-fold molar excess of IP_6_. TROSY^59^ and TROSY-based 3D HNCACB, HNCO, and HN(CA)CO triple resonance NMR spectra^60^ were recorded at 600 MHz while a 3D H(N)NH-NOESY-TROSY spectrum^61^ was recorded at 900 MHz with a NOESY mixing time of 100 ms. The NMR spectra were processed by PROSA^62^ and were analyzed by CARA and XEASY^63^. Secondary chemical shift plots were calculated by C_α_ and C_β_ values that considered random chemical shifts of every amino acid^64^ and amino acid specific deuterium isotope shift due to perdeuteration of the protein^65^. The assignment of amide resonances for SPX2 mutants was done by matching pairs of nearest peaks to the wild type, under consideration of structure and sequence information.

### Relaxation experiments

*T*_1_(^15^N), *T*_2_(^15^N) and hetNOE experiments^66^ were recorded on a 380 μM [*U*-^15^N]-labeled SPX2 sample at 600 MHz. The interscan delay used for *T*_1_ and *T*_2_ was 7.0 s and for hetNOE 5.3 s. Spectra were recorded with relaxation delays of 0–119 ms in steps of 17 ms for *T*_2_ and 0–1120 ms in steps of 160 ms for *T*_1_, processed with TopSpin 3.7 (*Bruker*) and fitted with exponential decay equations and covariance error method estimation in CCPNMR^67^.

### H/D exchange

SPX2 sample was exchanged from H_2_O- to D_2_O-containing by several buffer exchanges in an Amicon Ultra centrifugal filter (*Merck Millipore*) and was incubated in D_2_O-containing buffer for 12 h before recording H/D exchange. H/D exchange was estimated by intensity comparison of backbone amide peaks from 2D [^15^N,^1^H]-TROSY HSQC spectra of 200 μM [^2^H,^15^N,^13^C]-labeled SPX2 and of 200 μM [*U*-^2^H,^15^N,^13^C]-labeled SPX2 in the D_2_O containing buffer. The NMR spectra were acquired at 900 MHz in MST buffer (25 mM HEPES pH 7.0, 50 mM NaCl, 0.5 mM EDTA and 0.5 mM TCEP).

### Titration and chemical shift difference experiments

For the titration series, 2D [^15^N,^1^H]-TROSY spectra were obtained with 200 μM [*U*-^15^N]-labeled SPX2 under different buffer conditions at 600 MHz. Salt titration compared 2D [^15^N,^1^H]-TROSY spectra recorded at 50 mM NaCl and 350 mM NaCl, while IP_x_ titration compared 2D [^15^N,^1^H]-TROSY spectra recorded without and with 10-fold molar excess of IP_x_. 2D [^15^N,^1^H]-TROSY spectra of 500 μM [*U*-^2^H,^15^N]-labeled SPX2, SPX2∆α7 or SPX2∆linker were recorded at 600 MHz. The peak lists of two comparing states were extracted by CCPNMR and chemical shift perturbations were calculated in MATLAB R2017a using $\Delta \delta=\sqrt{({\delta_{1Rf}}({\,}^1H)-{\delta_{1}}({\,}^1H))^{2}+({\delta_{2Rf}}({\,}^{15}N)/5-\delta_{2}({\,}^{15}N)/5)^{2}}$. Titration curves were fitted to a Langmuir binding isotherm. For comparison of binding affinities between different IP_x_ to SPX domain, a titration series of 2D [^15^N,^1^H]-TROSY spectra were obtained on 400 μM [*U*-^15^N]-labeled SPX2 under different molar ratios of IP_x_ at 600 MHz.

### Binding experiments

2D [^15^N,^1^H]-TROSY spectra of 85 μM [*U*-^15^N]-labeled SPX2 were recorded in the presence and absence of 255 μM unlabeled Vtc4*. Another 2D [^15^N,^1^H]-TROSY spectrum was recorded after IP_6_ had been added in a 40-fold molar excess.

### RDC experiments

Bacteriophage pf1 (*ASLA Biotech*) was buffer exchanged by a few ultracentrifugation steps (95,000 *g* for 45 min at 4 °C in the TLA-100 *Beckman* rotor) and subsequently added to an SPX2 sample. 2D [^15^N,^1^H]-TROSY and 2D [^15^N,^1^H]-anti-TROSY spectrum was acquired of 200 μM of [^2^H, ^15^N]-labeled SPX2 and [^2^H, ^15^N]-labeled SPX2 that contained approximately 18 mg/mL bacteriophage causing quadrupolar deuterium splitting of 7 Hz. The NMR experiments were recorded at 900 MHz and residual dipolar couplings were calculated based on extracted ^1^*J*_NH_ coupling in aligned and unaligned sample by CCPNMR.

### Bioinformatic analysis

#### Homology modeling

The SPX2 protein sequence was submitted to the *Phyre2* Protein Fold Recognition server^68^ for structural modeling. The resulting model was superimposed with crystal structure of ligand-bound SPX4 (PDB 5IJP [10.2210/pdb5IJP/pdb]), to determine the expected position of a bound IP_6_ ligand. Secondary structure predictions for the region of helix α7 were obtained from the *Alphafold* models AF-P43585-F1 (Vtc2) and AF-Q02725-F1 (Vtc3)^44,45^.

#### Classification of SPX proteins by domain architecture

A list of SPX-containing protein sequences was obtained from the UniRef100 database, release 2020_05^69,70^. Architecture information for each sequence in the list was scraped from the InterPro database^71^, using a custom Python script and used to build a local SPX protein database (SPXdb). The generated SPXdb was filtered based on: (i) SPX domain position = 1; (ii) number of adjacent domains ≥ 1; (iii) SPX domain length ≥ 130 aa;^3^ and (iv) interdomain linker length ≤ 300 aa. Sequences in the filtered SPXdb were then grouped depending on the domain C-terminally adjacent to SPX.

#### Helix α7 motif generation and search

A list of protein sequences similar to Vtc2 from *S. cerevisiae* was obtained from Vtc2’s UniRef50 cluster (id: UniRef50_P43585) and aligned with Clustal-Omega^72^ with default parameters (Gonnet matrix, 6 bits gap opening penalty, 1 bit gap extension penalty). Sequences were removed from the list if 100% identical to other entries or if not aligning well with Vtc2 in the linker region. SPX’s helix α7 gap-less stretch was extracted from the alignment and used to generate a position-specific scoring matrix (PSSM) and a sequence logo^73,74^ using a custom Python script. The linker sequence of each entry of SPXdb was scanned with the generated PSSM. Hits were considered as such if the log-likelihood score exceeded 0.4 × log-likelihood(consensus sequence).

#### Protein stability measurements

The stability of protein samples was estimated by thermal denaturation (15–95 °C), monitored by nano differential scanning fluorimetry (nanoDSF) detecting the intrinsic fluorescence of tryptophane at 330 and 350 nm with a heating rate of 1 °C / min (Prometheus NT.48, *Nanotemper Technologies*). A total of 50 μM of SPX2 and 50 μM of SPX2∆α7 were loaded into standard capillaries and measured at 75% laser power. 200 μM of SPX2 alone as well as in the presence of a different ligand, IP_6_ or chemically stable 5-IP_7_ or 1,5-IP_8_, in a 10-fold molar excess were applied into standard capillaries and measured at 20% laser power. The measurements were conducted in triplicates in the NMR buffer (25 mM HEPES pH 7.0, 250 mM NaCl, 0.5 mM EDTA and 0.5 mM TCEP).

#### MicroScale thermophoresis binding affinities

Vtc2*, SPX2 and TTM2 proteins were buffer exchanged by multiple cycles of dilution in MST buffer (15 mM HEPES pH 7.0, 50 mM NaCl and 2 mM DTT) and subsequent ultrafiltration using Amicon ultracentrifugal filters with 10 kDa molecular weight cutoff. To test the effect of sodium chloride or IP_x_ molecules, the MST buffer was supplemented with additionally 100 mM NaCl or 2.5 mM of IP_x_ - IP_6_, natural 5-IP_7_, or 1,5-IP_8_. His-tagged proteins Vtc4* or TTM4 were diluted into MST buffer by ~400 times to obtain a 0.2 μM protein sample that was used for fluorescent labeling. It was mixed with 0.1 μM of RED-tris-NTA 2^nd^ generation dye (*Nanotemper Technologies*), incubated for 30 min at room temperature and subsequently centrifuged for 10 min at 21,000 *g* at 4 °C. For the Vtc4*–TTM2 experiment, Vtc4* was labeled with RED-NHS 2^nd^ generation dye (*Nanotemper Technologies*) following the manufacturer’s guidelines, buffer exchanged to MST buffer, and centrifuged for 10 min at 21,000 *g* at 4 °C. The fluorescent protein samples were mixed in a serial dilution with the protein titrant whose stock solution was in the low millimolar range, loaded into premium capillaries, and measured on Monolith NT.115 (*Nanotemper Technologies*) by 100 % laser power. Measurements were conducted in triplicates except for the two experiments shown in Supplementary Fig. 3e, which were conducted in duplicates due to limited protein material. The thermophoresis (F_norm_) of the first 1.5 s after laser irradiation was considered to avoid artifacts due to heating effects. MST experiments were fitted by F_norm_ with the exception of MST triplicates that were measured at higher salt concentrations. They had the same magnitude of thermophoresis change but revealed a small difference in the fluorescence baseline of free and bound state. To account for this, ∆F_norm_ was used for the fitting. The fitting of the thermophoresis data sets considered the information about the change of initial fluorescence intensity observed. The change of initial fluorescence intensity and thermophoresis monitor the same binding event as their evaluated dissociation constant *K*_D_ is in the same range and can be globally fitted as well. Dissociation constants *K*_D_ were determined by PRISM 8.4.3 software with the following equations:

1 $$\,\left[{{{{{\boldsymbol{PL}}}}}}\right]=\frac{({\left[{{{{{\boldsymbol{P}}}}}}\right]}_{{{{{{\bf{t}}}}}}}+{[{{{{{\boldsymbol{L}}}}}}]}_{{{{{{\bf{t}}}}}}}+{{{{{{\boldsymbol{K}}}}}}}_{{{{{{\bf{D}}}}}}})-\,\sqrt{{({[{{{{{\boldsymbol{P}}}}}}]}_{{{{{{\bf{t}}}}}}}+{\left[{{{{{\boldsymbol{L}}}}}}\right]}_{{{{{{\bf{t}}}}}}}+{{{{{{\boldsymbol{K}}}}}}}_{{{{{{\bf{D}}}}}}})}^{{{{{{\bf{2}}}}}}}-{{{{{\bf{4}}}}}}{[{{{{{\boldsymbol{P}}}}}}]}_{{{{{{\bf{t}}}}}}}{[{{{{{\boldsymbol{L}}}}}}]}_{{{{{{\bf{t}}}}}}}}}{{{{{{\bf{2}}}}}}}.$$

2 $$[{{{{{\boldsymbol{P}}}}}}]={[{{{{{\boldsymbol{P}}}}}}]}_{{{{{{\bf{t}}}}}}}-\left[{{{{{\boldsymbol{PL}}}}}}\right].$$

3 $${{{{{{\boldsymbol{S}}}}}}}_{{{{{{\bf{obs}}}}}}}=\frac{[{{{{{\boldsymbol{P}}}}}}]\cdot {{{{{{\boldsymbol{\varepsilon }}}}}}}_{{{{{{\bf{P}}}}}}}\cdot {{{{{{\boldsymbol{\alpha }}}}}}}_{{{{{{\bf{P}}}}}}}+[{{{{{\boldsymbol{PL}}}}}}]\cdot {{{{{{\boldsymbol{\varepsilon }}}}}}}_{{{{{{\bf{PL}}}}}}}\cdot {{{{{{\boldsymbol{\alpha }}}}}}}_{{{{{{\bf{PL}}}}}}}}{[{{{{{\boldsymbol{P}}}}}}]\cdot {{{{{{\boldsymbol{\varepsilon }}}}}}}_{{{{{{\bf{P}}}}}}}+[{{{{{\boldsymbol{PL}}}}}}]\cdot {{{{{{\boldsymbol{\varepsilon }}}}}}}_{{{{{{\bf{PL}}}}}}}}$$

where [*P*]_t_ is the total concentration of His-tagged protein (Vtc4* or TTM4) and [*L*]_*t*_ the total concentration of Vtc2* or SPX protein for a given titration point. *S*_obs_ is the observed *F*_norm_ signal, where *α*_P_ and *α*_PL_ are the *F*_norm_ signals for free and bound protein state, while *ε*_P_ and *ε*_PL_ are their relative or absolute fluorescence intensities. The latter are fixed parameters obtained from the fluorescence intensity data.

#### Genetic manipulation of yeast strains

Strain BY4742 vtc2::LEU2 vtc3::natNT2 vtc4::kanMX was made based on BY4742 vtc4::kanMX (*Euroscarf*) by replacing the entire open reading frames of VTC2 and VTC3 genes with a corresponding marker cassette^75^. The strain is described in ref. ^8^. VTC3 and VTC4 alleles under the control of endogenous promoters were cloned into pRS306 and pRS303 plasmids, respectively, which were then integrated into the genome. The resulting mutants carry substitutions in corresponding residues of the SPX domains of VTC3 and VTC4, which are conserved between VTC2, VTC3, and VTC4. They do not express a VTC2-containing complex, which facilitates the analysis. VTC protein levels on isolated vacuoles were verified by Western blotting (Antibodies to Vtc2 generated in rabbits by Eurogentec, used at 1:150 dilution in PBS with 5% milk powder. IRDye 800CW Goat anti-Rabbit IgG (H + L) N° 926-32211 used at a dilution of 1:1000). Protein levels ranged between 80–120% of the levels in vtc2Δ “wild-type”, which had been reconstituted with VTC3 and VTC4.

#### Co-immunoadsorption

BY 4741 ∆pep4 ∆prb1 yeast cells were transfected with a plasmid expressing Vtc4SPX domain (aa 1–294) with a Gly_6_ linker and a 3xFLAG from an ADH promotor (pRS416-pADH-SPXvtc4(1–294)-Gly_6_FLAG_3_). The strain is described in ref. ^8^. The cells were logarithmically grown in SC-URA medium overnight. They were harvested at an OD_600_ of 1 by centrifugation (5 min, 3,000 *g*, 4 °C). The pellet was washed once in cold TGN buffer (5% glycerol, 100 mM NaCl, 50 mM Tris/Cl pH 7.4), centrifuged as before, resuspended in 500 µl of IP buffer (0.5% Tween 20, 1 mM DTT, 1x protease inhibitor cocktail (100 µM Pefabloc SC protease inhibitor (Roth, cat. no. A154.2), 0.1 µg/ml Leupeptin (Bachem, cat. no. N1000.0005), 100 µM o-phenantrolin-hydrochloride (Millipore, cat. no. 516705), 0.5 µg/ml Pepstatin A (Bachem, cat. no. N1125.0025), 1 mM PMSF (prepared freshly as 200 mM stock in isopropanol and added right before use), in TGN buffer) and transferred into two 2 ml Eppendorf safe-lock tubes/strain. 400 µl glass beads were added per tube and the samples were vortexed for 10–15 min at maximal speed in the cold room. Glass beads were allowed to sediment. The lysed cells were pooled in a 2 ml Eppendorf tube and centrifuged (16,000 *g*, 4 °C, 10 min). The supernatant was recovered, and protein content was estimated in a nanodrop spectrophotometer via the OD_280_. The sample volume was adjusted to 500 µl with IP buffer. 20 µl of this extract were withdrawn to serve as the input control. They were mixed with 10 µl IP buffer and 10 µl 4xNuPage buffer with DTT, and heated immediately for 10 min at 70 °C. 50 µl per sample anti Flag-Dynabeads (Dynabeads™ Protein G for Immunoprecipitation (Invitrogen-ThermoFisher, cat. no. 10004D) coupled with Monoclonal ANTI-FLAG® M2 antibody produced in mouse (Sigma-Merck, cat. no. F1804) were rinsed 3 times with IP buffer and all liquid was withdrawn from the beads. Beads were resuspended in 50 µl IP buffer, added to the sample and gently mixed on a rotating wheel at 4 °C for 1.5–2 h. Then, the tubes were briefly centrifuged and then put on magnetic rack. 20 µl of the supernatant were withdrawn (flowthrough control), supplemented with 10 µl IP buffer and 10 µl 4x NuPage buffer with DTT, and heated immediately for 10 min at 70 °C. The rest of the supernatant was removed, the beads were transferred to a new tube, washed three times with the same IP buffer and transferred into new tubes. Finally, the Dynabeads were resuspended in a total volume of 100 µl containing 0.8 µM purified recombinant Vtc2-SPX domain with or without 10 µM 1.5-P_8_, and the samples were again incubated on a rotating wheel for 1.5 h at 4 °C. The beads were sedimented and washed 3 times with 1 ml IP buffer. With the last washing step, the beads were transferred to a new tube, sedimented again and the supernatant was discarded. The beads were eluted with 30 µl IP buffer and 10 µl 4xNuPage/DTT buffer, heated (5 min, 95 °C) and the supernatant was loaded in NuPAGE gels. The uncropped gel is shown in Supplementary Fig. 3b.

#### Crosslinking experiments

30 mL of reaction containing 80 μM of SPX2 and 80 μM Vtc4* in the presence or absence of 20-molar excess of IP_6_, as well as negative controls comprising either 80 μM of SPX2 and 80 μM or Vtc4ΔTM, respectively, were kept on ice. The samples were put at room temperature for 5 min and 2 mM 1,1’-carbonyldiimidazole (CDI, *Sigma-Aldrich*) stored in dimethyl sulfoxide (DMSO, *Sigma-Aldrich*) was added. The reaction was quenched by 500 mM Tris, pH 8.0 solution after 30 s. The reaction was performed in cross-link buffer (25 mM HEPES pH 7.0, 150 mM NaCl, 0.5 mM EDTA, 0.5 mM TCEP). The uncropped gel is shown in Supplementary Fig. 8a.

#### VTC activity assay

Polyphosphate synthesis activity of the VTC complex was assayed in vitro^8^. Vacuole isolation: Cells were logarithmically grown in 1 L YPD media at 30 °C overnight. Cells were harvested at OD_600nm_ of 1–2 and vacuoles were isolated as described^31^. Vacuole protein concentrations were determined by Bradford assay, using fatty acid-free BSA as a standard. To follow the reaction time-course, 0.005 mg/ml vacuoles were incubated in 1 mL reaction buffer (10 mM PIPES/KOH pH 6.8, 150 mM KCl, 0.5 mM MnCl_2_, 200 mM sorbitol), containing an ATP-regenerating system (1 mM ATP-MgCl_2_, 20 mM creatine phosphate and 0.25 mg/ml creatine kinase) at 27 °C. Where indicated, 1 µM 5-InsP_7_ was added during the incubation. At the indicated time points, 80 µl aliquots were withdrawn and mixed with 160 µl of stop solution (10 mM PIPES/KOH pH 6.8, 150 mM KCl, 200 mM sorbitol, 12 mM EDTA, 0.15 % (v/v) Triton X-100 and 15 µM DAPI) in a black 96-well plate. After equilibration for 15 min in the dark, which allows the binding of DAPI to polyP, the fluorescence of the polyP-DAPI complex was measured with a SPECTRAmax GEMINI XS (*Molecular Devices*) fluorescence plate reader (λ_ex_ = 415 nm, λ_em_ = 550 nm, cutoff = 530 nm, 27 °C). Synthesized polyP was calculated from a calibration curve prepared with synthetic polyP 60 (*Sigma-Aldrich*).

### Reporting summary

Further information on research design is available in the Nature Portfolio Reporting Summary linked to this article.

## Supplementary information

Supplementary Information

Reporting Summary

## Data Availability

The NMR chemical shift assignments of SPX2 have been deposited in the BMRB data base with accession code 51877. The experimental data that support the findings of this study are shown in the article and its supplementary materials. The raw data underlying all Figures and Supplementary Figures are provided as a Source Data file. Any additional information required to reanalyze the data reported in this paper will be shared by the corresponding author upon request. PDB structures were accessed at https://www.rcsb.org/: 5IIG (Apo SPX4 – form A) and 5IIQ (Apo SPX4 – form B), 5IJP (holo SPX4), 5IJH) (human Xpr1), and 5IJJ) (Gde1). The data base UniRef100 was accessed at https://www.uniprot.org/, the sequences assessed are given in Supplementary Table 1. Source data are provided with this paper.

## Source data

Source Data

## References

[1] Austin S, Mayer A (2020). Phosphate homeostasis - a vital metabolic equilibrium maintained through the INPHORS signaling pathway. Front. Microbiol..

[2] Secco D, Wang C, Shou H, Whelan J (2012). Phosphate homeostasis in the yeast Saccharomyces cerevisiae, the key role of the SPX domain-containing proteins. FEBS Lett..

[3] Secco D (2012). The emerging importance of the SPX domain-containing proteins in phosphate homeostasis. N. Phytol..

[4] Hürlimann HC, Pinson B, Stadler-Waibel M, Zeeman SC, Freimoser FM (2009). The SPX domain of the yeast low-affinity phosphate transporter Pho90 regulates transport activity. EMBO Rep..

[5] Legati A (2015). Mutations in XPR1 cause primary familial brain calcification associated with altered phosphate export. Nat. Genet..

[6] López-Sánchez U (2020). Interplay between primary familial brain calcification-associated SLC20A2 and XPR1 phosphate transporters requires inositol polyphosphates for control of cellular phosphate homeostasis. J. Biol. Chem..

[7] Li X (2020). Control of XPR1-dependent cellular phosphate efflux by InsP8 is an exemplar for functionally-exclusive inositol pyrophosphate signaling. Proc. Natl Acad. Sci. USA.

[8] Wild R (2016). Control of eukaryotic phosphate homeostasis by inositol polyphosphate sensor domains. Science.

[9] Puga MI (2014). SPX1 is a phosphate-dependent inhibitor of Phosphate Starvation Response 1 in Arabidopsis. Proc. Natl Acad. Sci. USA.

[10] Liu F (2010). OsSPX1 suppresses the function of OsPHR2 in the regulation of expression of OsPT2 and phosphate homeostasis in shoots of rice. Plant J..

[11] Wang Z (2014). Rice SPX1 and SPX2 inhibit phosphate starvation responses through interacting with PHR2 in a phosphate-dependent manner. Proc. Natl Acad. Sci. USA.

[12] Qi W, Manfield IW, Muench SP, Baker A (2017). AtSPX1 affects the AtPHR1-DNA-binding equilibrium by binding monomeric AtPHR1 in solution. Biochem. J..

[13] Ried MK (2021). Inositol pyrophosphates promote the interaction of SPX domains with the coiled-coil motif of PHR transcription factors to regulate plant phosphate homeostasis. Nat. Commun..

[14] Battini JL, Rasko JE, Miller AD (1999). A human cell-surface receptor for xenotropic and polytropic murine leukemia viruses: possible role in G protein-coupled signal transduction. Proc. Natl Acad. Sci. USA.

[15] Potapenko E (2018). 5-Diphosphoinositol pentakisphosphate (5-IP7) regulates phosphate release from acidocalcisomes and yeast vacuoles. J. Biol. Chem..

[16] Gerasimaitė R (2017). Inositol pyrophosphate specificity of the SPX-dependent polyphosphate polymerase VTC. ACS Chem. Biol..

[17] Wild R, Hothorn M (2017). The macro domain as fusion tag for carrier-driven crystallization. Protein Sci..

[18] Desmarini D (2020). IP7-SPX domain interaction controls fungal virulence by stabilizing phosphate signaling machinery. mBio.

[19] Xu B (2012). De novo gene mutations highlight patterns of genetic and neural complexity in schizophrenia. Nat. Genet..

[20] Yao X-P (2017). Analysis of gene expression and functional characterization of XPR1: a pathogenic gene for primary familial brain calcification. Cell Tissue Res..

[21] Anheim M (2016). XPR1 mutations are a rare cause of primary familial brain calcification. J. Neurol..

[22] López-Sánchez U (2019). Characterization of XPR1/SLC53A1 variants located outside of the SPX domain in patients with primary familial brain calcification. Sci. Rep..

[23] Bondeson DP (2022). Phosphate dysregulation via the XPR1-KIDINS220 protein complex is a therapeutic vulnerability in ovarian cancer. Nat. Cancer.

[24] Nicolas G (2013). Phenotypic spectrum of probable and genetically-confirmed idiopathic basal ganglia calcification. Brain.

[25] Dong J (2019). Inositol pyrophosphate InsP8 acts as an intracellular phosphate signal in Arabidopsis. Mol. Plant.

[26] Lv Q (2014). SPX4 negatively regulates phosphate signaling and homeostasis through Its interaction with PHR2 in rice. Plant Cell.

[27] Zhong Y (2018). Rice SPX6 negatively regulates the phosphate starvation response through suppression of the transcription factor PHR2. N. Phytol..

[28] Zhu J (2019). Two bifunctional inositol pyrophosphate kinases/phosphatases control plant phosphate homeostasis. eLife.

[29] Desfougères Y, Gerasimaitė RU, Jessen HJ, Mayer A (2016). Vtc5, a novel subunit of the vacuolar transporter chaperone complex, regulates polyphosphate synthesis and phosphate homeostasis in yeast. J. Biol. Chem..

[30] Müller O (2002). The Vtc proteins in vacuole fusion: coupling NSF activity to V(0) trans-complex formation. EMBO J..

[31] Gerasimaitė R, Sharma S, Desfougères Y, Schmidt A, Mayer A (2014). Coupled synthesis and translocation restrains polyphosphate to acidocalcisome-like vacuoles and prevents its toxicity. J. Cell. Sci..

[32] Hothorn M (2009). Catalytic core of a membrane-associated eukaryotic polyphosphate polymerase. Science.

[33] Guan Z (2023). The cytoplasmic synthesis and coupled membrane translocation of eukaryotic polyphosphate by signal-activated VTC complex. Nat. Commun..

[34] Liu, W. et al. Cryo-EM structure of the polyphosphate polymerase VTC: Coupling polymer synthesis to membrane transit. *bioRxiv*10.1101/2023.01.27.525886 (2023). 10.15252/embj.2022113320 PMC10183816 37066886

[35] Cohen A, Perzov N, Nelson H, Nelson N (1999). A novel family of yeast chaperons involved in the distribution of V-ATPase and other membrane proteins. J. Biol. Chem..

[36] Nelson N (2000). The cellular biology of proton-motive force generation by V-ATPases. J. Exp. Biol..

[37] Müller O, Neumann H, Bayer MJ, Mayer A (2003). Role of the Vtc proteins in V-ATPase stability and membrane trafficking. J. Cell Sci..

[38] Albert C (1997). Biological variability in the structures of diphosphoinositol polyphosphates in Dictyostelium discoideum and mammalian cells. Biochem. J..

[39] Barker CJ, Wright J, Hughes PJ, Kirk CJ, Michell RH (2004). Complex changes in cellular inositol phosphate complement accompany transit through the cell cycle. Biochem. J..

[40] Wundenberg T, Mayr GW (2012). Synthesis and biological actions of diphosphoinositol phosphates (inositol pyrophosphates), regulators of cell homeostasis. Biol. Chem..

[41] Illies C (2007). Requirement of inositol pyrophosphates for full exocytotic capacity in pancreatic beta cells. Science.

[42] Lin H (2009). Structural analysis and detection of biological inositol pyrophosphates reveal that the family of VIP/diphosphoinositol pentakisphosphate kinases are 1/3-kinases. J. Biol. Chem..

[43] Chabert, V. et al. Inositol pyrophosphate dynamics in yeast reveals control of the PHO starvation program through 1,5-IP_8_ and the SPX domain of the CDK inhibitor Pho81. *bioRxiv*10.1101/2023.02.14.528555 (2023). 10.7554/eLife.87956 PMC10511240 37728314

[44] Jumper J (2021). Highly accurate protein structure prediction with AlphaFold. Nature.

[45] Varadi M (2022). AlphaFold protein structure database: massively expanding the structural coverage of protein-sequence space with high-accuracy models. Nucleic Acids Res..

[46] Ogawa N, DeRisi J, Brown PO (2000). New components of a system for phosphate accumulation and polyphosphate metabolism in *Saccharomyces cerevisiae* revealed by genomic expression analysis. Mol. Biol. Cell.

[47] Capolicchio S, Wang H, Thakor DT, Shears SB, Jessen HJ (2014). Synthesis of densely phosphorylated bis-1,5-diphospho-*myo*-inositol tetrakisphosphate and its enantiomer by bidirectional P-anhydride formation. Angew. Chem. Int. Ed..

[48] Riemer E (2021). ITPK1 is an InsP6/ADP phosphotransferase that controls phosphate signaling in Arabidopsis. Mol. Plant.

[49] Gu C (2017). The significance of the bifunctional kinase/phosphatase activities of diphosphoinositol pentakisphosphate kinases (ppip5ks) for coupling inositol pyrophosphate cell signaling to cellular phosphate homeostasis. J. Biol. Chem..

[50] Holt LJ (2009). Global analysis of Cdk1 substrate phosphorylation sites provides insights into evolution. Science.

[51] MacGilvray ME (2020). Phosphoproteome response to dithiothreitol reveals unique versus shared features of *Saccharomyces cerevisiae* stress responses. J. Proteome Res..

[52] Swaney DL (2013). Global analysis of phosphorylation and ubiquitylation cross-talk in protein degradation. Nat. Methods.

[53] Lanz MC (2021). In-depth and 3-dimensional exploration of the budding yeast phosphoproteome. EMBO Rep..

[54] Albuquerque CP (2008). A multidimensional chromatography technology for in-depth phosphoproteome analysis. Mol. Cell. Proteom..

[55] Soulard A (2010). The rapamycin-sensitive phosphoproteome reveals that TOR controls protein kinase A toward some but not all substrates. Mol. Biol. Cell.

[56] Wu W (2013). MicroRNA-18a modulates STAT3 activity through negative regulation of PIAS3 during gastric adenocarcinogenesis. Br. J. Cancer.

[57] Riley AM, Wang H, Shears SB, Potter L (2015). B. V. Synthetic tools for studying the chemical biology of InsP8. Chem. Commun..

[58] Hager A (2016). Cellular cations control conformational switching of inositol pyrophosphate analogues. Chem. – A Eur. J..

[59] Pervushin K, Riek R, Wider G, Wüthrich K (1997). Attenuated *T*_2_ relaxation by mutual cancellation of dipole-dipole coupling and chemical shift anisotropy indicates an avenue to NMR structures of very large biological macromolecules in solution. Proc. Natl Acad. Sci. USA.

[60] Salzmann M, Pervushin K, Wider G, Senn H, Wüthrich K (1998). TROSY in triple-resonance experiments: new perspectives for sequential NMR assignment of large proteins. Proc. Natl Acad. Sci. USA.

[61] Xia Y, Sze K, Zhu G (2000). Transverse relaxation optimized 3D and 4D 15N/15N separated NOESY experiments of 15N labeled proteins. J. Biomolecular NMR.

[62] Güntert P, Dötsch V, Wider G, Wüthrich K (1992). Processing of multi-dimensional NMR data with the new software PROSA. J. Biomolecular NMR.

[63] Bartels C, Xia T, Billeter M, Güntert P, Wüthrich K (1995). The program XEASY for computer-supported NMR spectral analysis of biological macromolecules. J. Biomolecular NMR.

[64] Wishart DS, Bigam CG, Holm A, Hodges RS, Sykes BD (1995). 1H, 13C and 15N random coil NMR chemical shifts of the common amino acids. I. Investigations of nearest-neighbor effects. J. Biomolecular NMR.

[65] Venters RA, Farmer BT, Fierke CA, Spicer LD (1996). Characterizing the use of perdeuteration in nmr studies of large proteins:13c,15n and1h assignments of human carbonic anhydrase II. J. Mol. Biol..

[66] Zhu G, Xia Y, Nicholson LK, Sze KH (2000). Protein dynamics measurements by trosy-based nmr experiments. J. Magn. Reson.

[67] Vranken WF (2005). The CCPN data model for NMR spectroscopy: development of a software pipeline. Proteins.

[68] Kelley LA, Mezulis S, Yates CM, Wass MN, Sternberg MJE (2015). The Phyre2 web portal for protein modeling, prediction and analysis. Nat. Protoc..

[69] Consortium TU (2019). UniProt: a worldwide hub of protein knowledge. Nucleic Acids Res..

[70] Suzek BE (2015). UniRef clusters: a comprehensive and scalable alternative for improving sequence similarity searches. Bioinformatics.

[71] Blum M (2021). The InterPro protein families and domains database: 20 years on. Nucleic Acids Res..

[72] Madeira F (2019). The EMBL-EBI search and sequence analysis tools APIs in 2019. Nucleic Acids Res..

[73] Schneider TD, Stephens RM (1990). Sequence logos: a new way to display consensus sequences. Nucleic Acids Res..

[74] Tareen A, Kinney JB (2020). Logomaker: beautiful sequence logos in Python. Bioinformatics.

[75] Janke C (2004). A versatile toolbox for PCR-based tagging of yeast genes: new fluorescent proteins, more markers and promoter substitution cassettes. Yeast.

[76] Kyte J, Doolittle RF (1982). A simple method for displaying the hydropathic character of a protein. J. Mol. Biol..

