# Supplementary material for: Inositol pyrophosphates activate the vacuolar transport chaperone complex in yeast by disrupting a homotypic SPX domain interaction
Source: Nat Commun. 2023 May 8;14:2645. doi: 10.1038/s41467-023-38315-w (PMC10167327; doi:10.1038/s41467-023-38315-w)
Supplement: Supplementary file 1 — Supplementary Information [file 41467_2023_38315_MOESM1_ESM.pdf]

Supplementary Figures

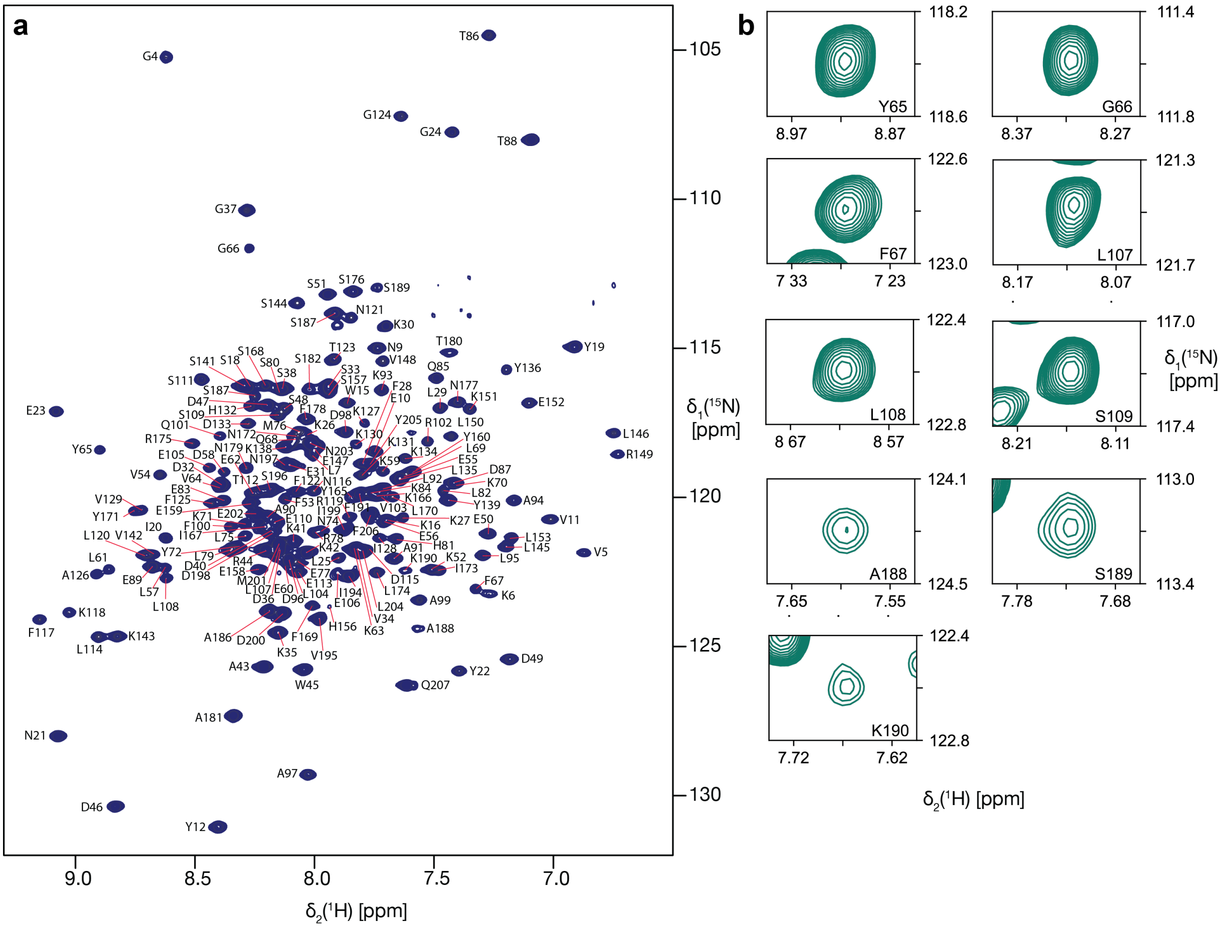

Supplementary Figure 1. NMR spectroscopy and assignments of SPX2. (**a**) 2D [^{15}N,^{1}H]-TROSY experiment of [*U*-^{2}H,^{15}N]-SPX2 in NMR buffer. Sequence-specific resonance assignments are indicated. (**b**) Zoom-in on the three resonance triplets from helices α3 (Y65–F67), α4 (L107–S109) and α7 (A188–K190), plotted at the same contour levels. The resonances of helix α7 have significantly reduced signal intensity compared to the others, indicating increased water exchange along with increased local dynamics.

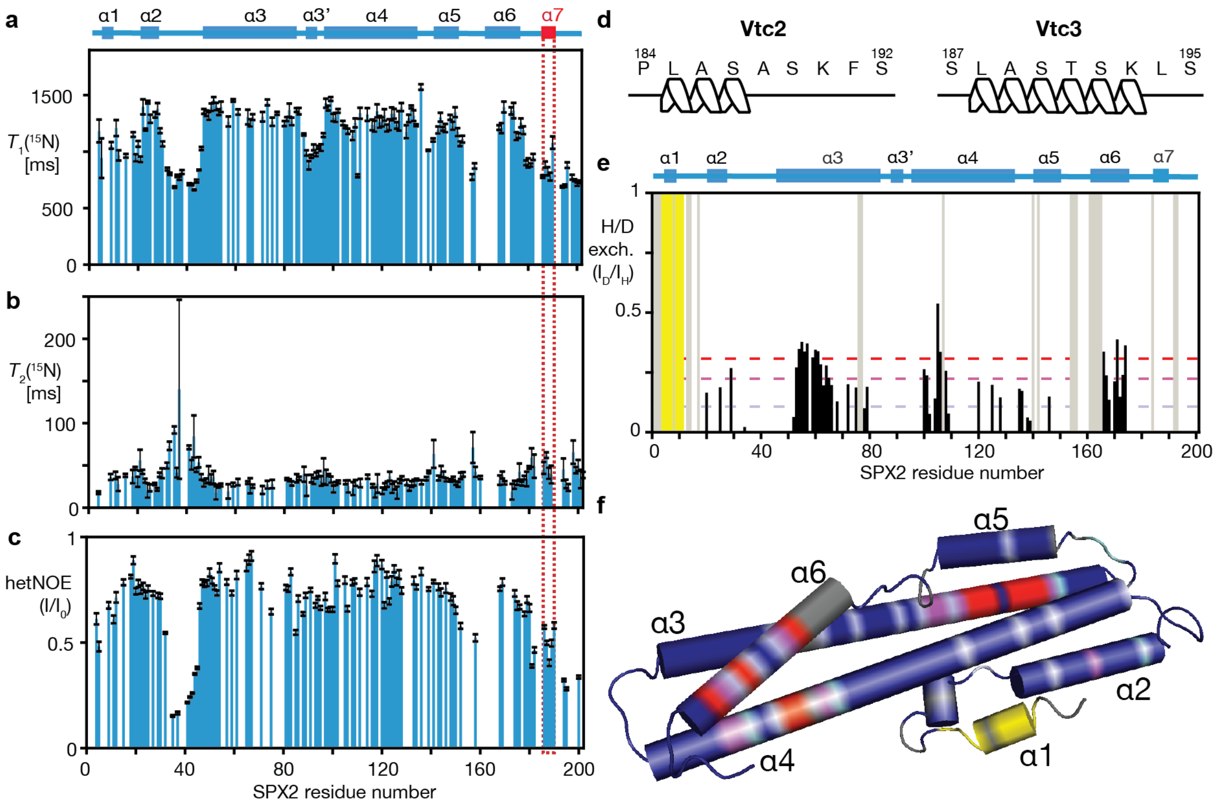

Supplementary Figure 2. Characterization of SPX2 backbone dynamics by NMR spectroscopy. (**a, b, c**) NMR spin relaxation parameters $T_1(^{15}N)$, $T_2(^{15}N)$ and hetNOE of SPX2 in NMR buffer, measured at 600 MHz field strength. Red dashed lines indicate the position of helix α7. Data were fitted from n = 8 relaxation time points. Error bars indicate fit error estimated by covariance method. (**d**) Secondary structure elements in Alphafold predictions for Vtc2 and Vtc3. Curl – helix; line – random coil. (**e**) Residue-specific H/D exchange of SPX2. I_D – intensities of resonances measured in D_2O-based MST buffer, I_H – intensities of resonances measured in H_2O-based MST buffer. Dashed horizontal lines (red/purple/light blue), indicate three threshold levels based on standard deviation for the classification of residues into high/medium/low exchange protection. (**f**) Protection against amide exchange plotted on a structural model of SPX2, as determined in D. Red/purple/light blue – high/medium/low protection; dark blue – no protection; yellow – residues experiencing intermediate chemical exchange; gray – residues not assigned. Source data are provided as a Source Data file.

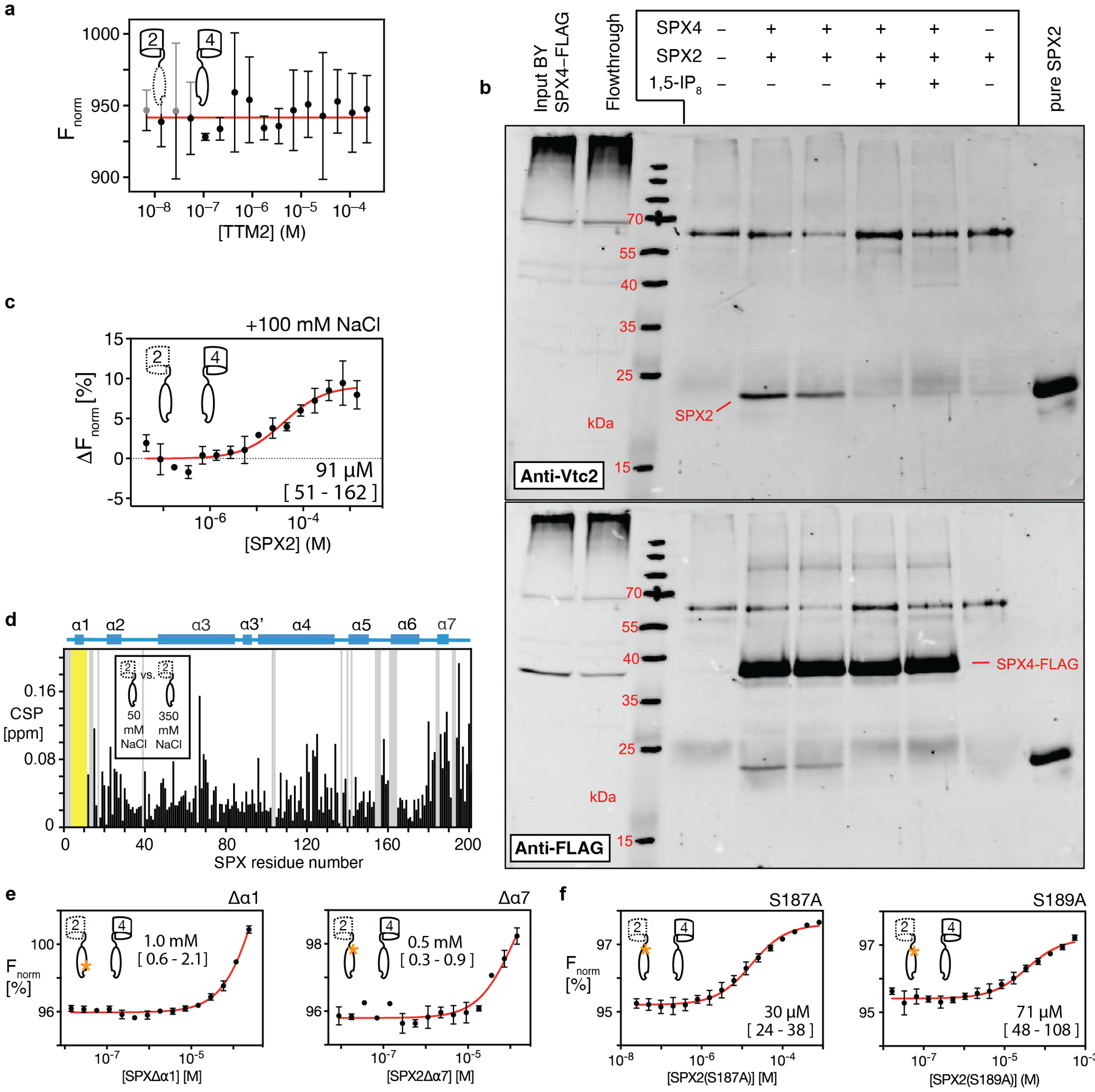

Supplementary Figure 3. Characterization of the binding between TTM2, SPX2 and Vtc4 constructs. (**a**) MST binding experiment of TTM2 to Vtc4*. No binding is detected, as indicated by the flat fitting curve (red). *n*=3 replicates. (**b**) Co-immunoadsorption of the SPX4 and SPX2 domains. The FLAG-tagged SPX4 domain was expressed as a soluble protein in yeast strain BY4741 Δpep4 Δprb1. A whole-cell extract expressing SPX4-FLAG or an empty plasmid was incubated with anti-FLAG-antibodies covalently linked to magnetic beads. The beads were washed, incubated with purified recombinant SPX2 in the presence or absence of 10 µM 1,5-IP_8, as indicated, washed again and analyzed by Western blotting, using antibodies to SPX2 (top blot) and FLAG (bottom blot). The experiment was performed *n*=3 times with similar results. (**c**) MST binding affinities of SPX2 to Vtc4* at elevated NaCl conditions. *n*=3 replicates. (**d**) Chemical shift perturbation plot of SPX2 induced by sodium chloride titration. Yellow -

residues experiencing intermediate chemical exchange, gray – residues not assigned. (**e**) Binding affinities of SPX2Δα1 or SPX2Δα7, respectively, to Vtc4* measured by MST. Orange asterisks display the protein lacking areas in the cartoon scheme. *n*=2 replicates. (**f**) MST binding affinities of SPX2 with single mutations to Vtc4* in the MST buffer. Asterisks show the mutated positions in the cartoon scheme. Graphs show the means and SEM; *n*=3 replicates. The dissociation constants determined by the fits are displayed along with a 95% confidence interval. Source data are provided as a Source Data file.

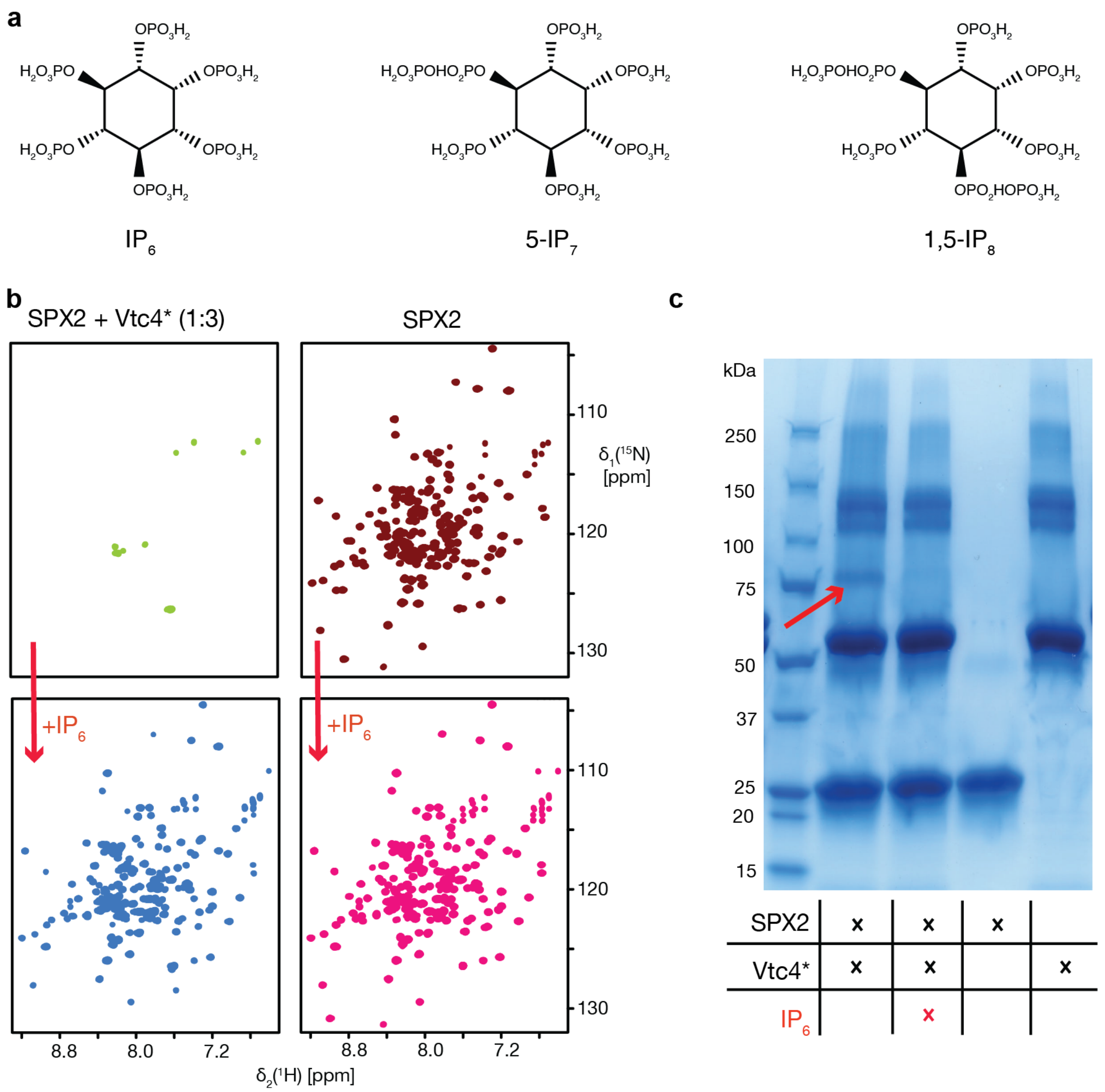

Supplementary Figure 4. IP$_6$ disrupts the interaction between SPX2 and Vtc4*. (**a**) Chemical structures of inositol phosphate (IP$_6$), 5-diphospho-inositol tetrakisphosphate (5-IP$_7$), 1,5-bis-diphospho-inositol tetrakisphosphate (1,5-IP$_8$). (**b**) NMR spectra of [*U*-^{15}N]-SPX2 and unlabeled Vtc4* recorded in a molar ratio 1:3 in the absence and presence of a 40x molar excess of IP$_6$. (**c**) Cross-linking experiment of SPX2 binding to Vtc4* with the cross-linker CDI in absence (1st lane) or presence of 20x molar excess of IP$_6$ (2nd lane). Negative controls (3rd and 4th lane) are single components cross-linked in the absence of a IP$_6$. The buffer used was 25 mM HEPES pH 7.0, 150 mM NaCl, 0.5 mM EDTA, 0.5 mM TCEP. The red arrow indicates a band with the expected size of a cross-linked SPX2–Vtc4* complex. The experiment was conducted once.

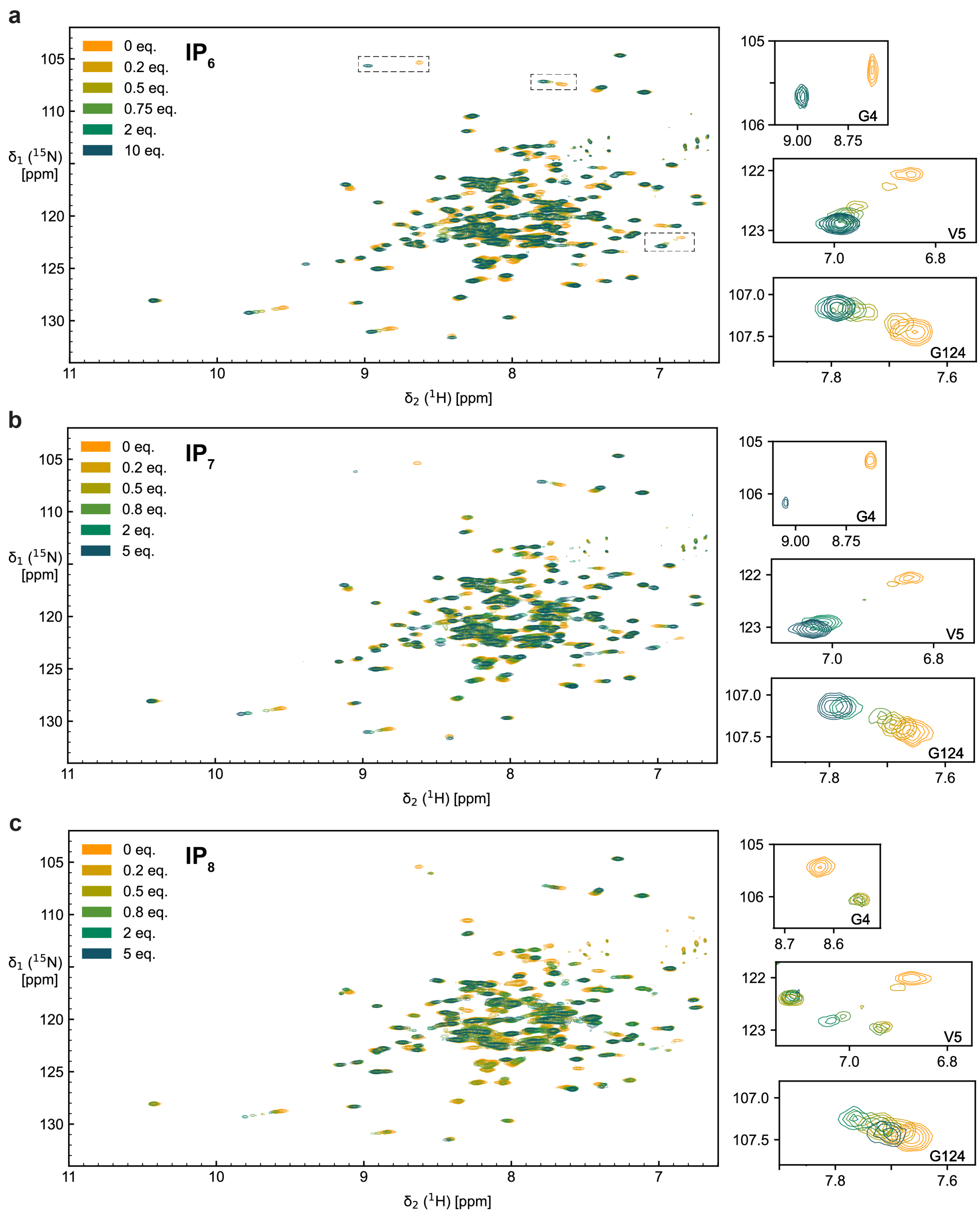

Supplementary Figure 5. NMR titration series of SPX2 with IP_x. Superimposed 2D [^{15}N,^{1}H]-TROSY spectra with different concentrations of IP_x, as indicated and zooms of three selected residues undergoing intermediate exchange. NMR titration was performed using (**a**) inositol phosphate (IP_6), (**b**) 5-diphospho-inositol tetrakisphosphate (5-IP_7), (**c**) 1,5-bis-diphospho-inositol tetrakisphosphate (1,5-IP_8) and [*U*-^{15}N]-SPX2 in NMR Buffer with 250mM NaCl.

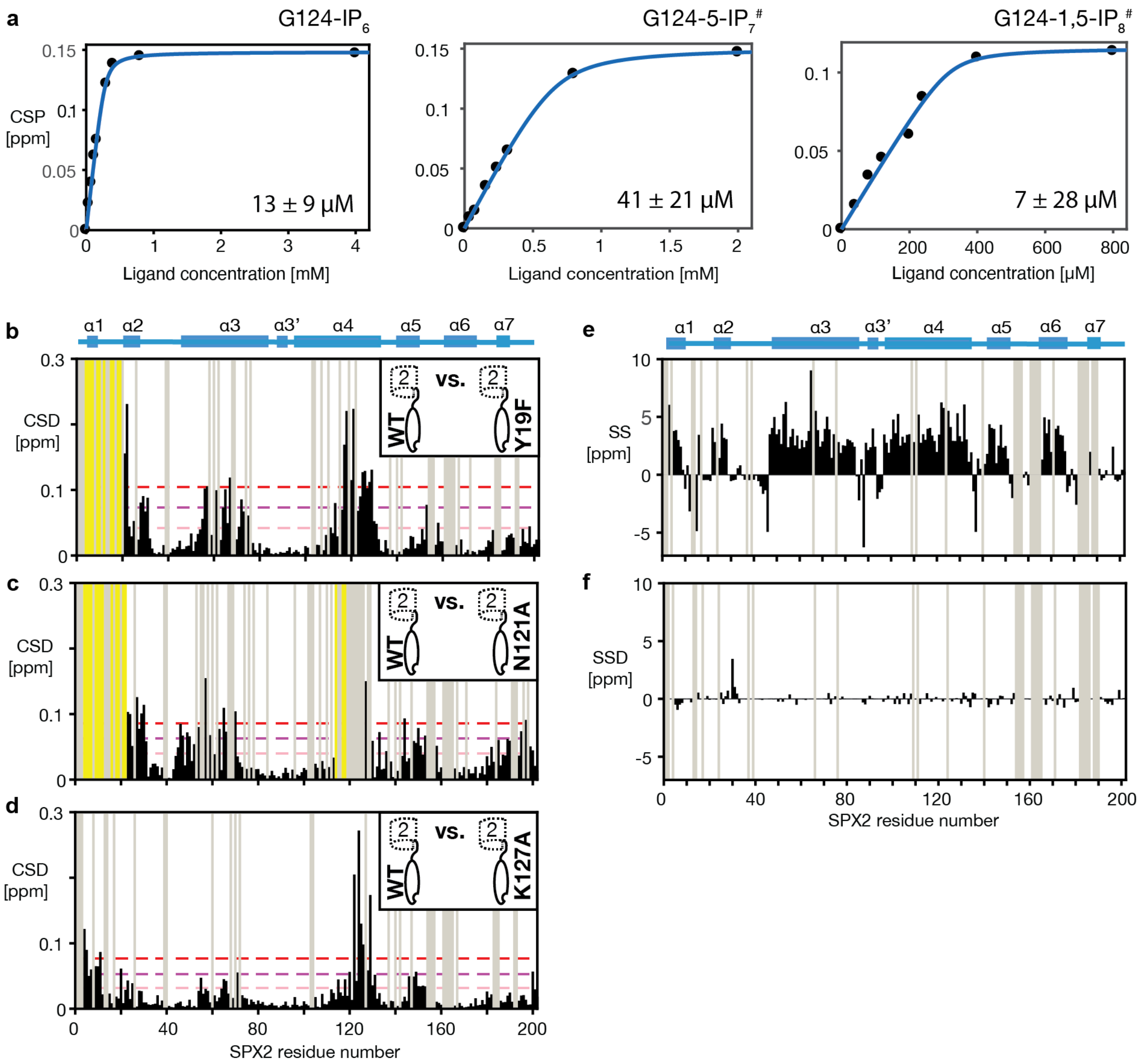

Supplementary Figure 6. Structural characterization of holo-state and pseudo-active mutants of SPX2. (**a**) Determination of dissociation constants of SPX2 bound to different non-hydrolysable IP_x by NMR on the example of glycine 124. Graphs show the means and SEM; *n*=3. (**b, c, d**) Chemical shift differences between the wild-type SPX2 and single-point mutations of SPX2 (Y19F, N121A, K127A). Yellow - residues experiencing intermediate chemical exchange, gray – residues not assigned. Dashed horizontal lines (red/purple/magenta), indicate three threshold levels based on standard deviation for the classification of residues into high/medium/low perturbation. (**e, f**) Secondary elements determination by secondary chemical shift (SS) plots of SPX2 in the presence of 10-fold molar excess of IP_6 and SS plot displaying differences between SS value in apo- and holo-state. Source data are provided as a Source Data file.

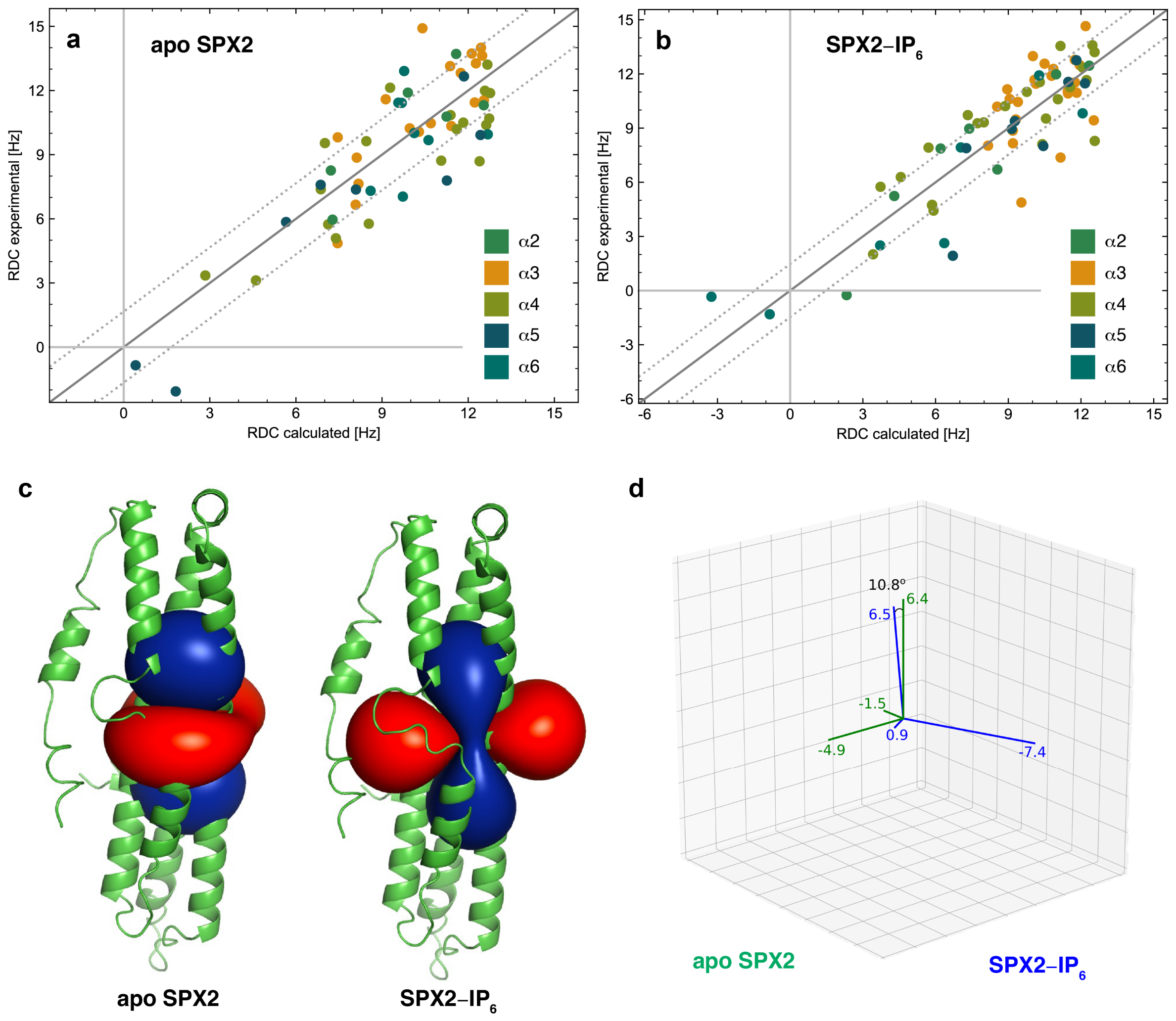

Supplementary Figure 7. Alignment of SPX2 in bacteriophage solution measured by residual dipolar couplings (RDCs). (**a**) Experimental versus calculated residual dipolar couplings of apo SPX2 in bacteriophage solution. Data points are colored per helix, as indicated. (**b**) Same for SPX2 with bound IP_6. (**c**) Display of the alignment tensors from the fits in A and B, on the structural model of SPX2, representing the value of a virtual N-H bond vector pointing in a particular direction of space. The red/blue isosurfaces represent the preferred/non-preferred orientation of the magnetic field with respect to the fixed protein frame. (**d**) Visualization of the alignment tensors in the coordinate system of the apo-SPX2 tensor. The axes are defined by three eigenvectors scaled by the corresponding eigenvalues in units of 10^{-32} m^3. The orientation of the tensor along the Z-axis is only slightly changed. Source data are provided as a Source Data file.

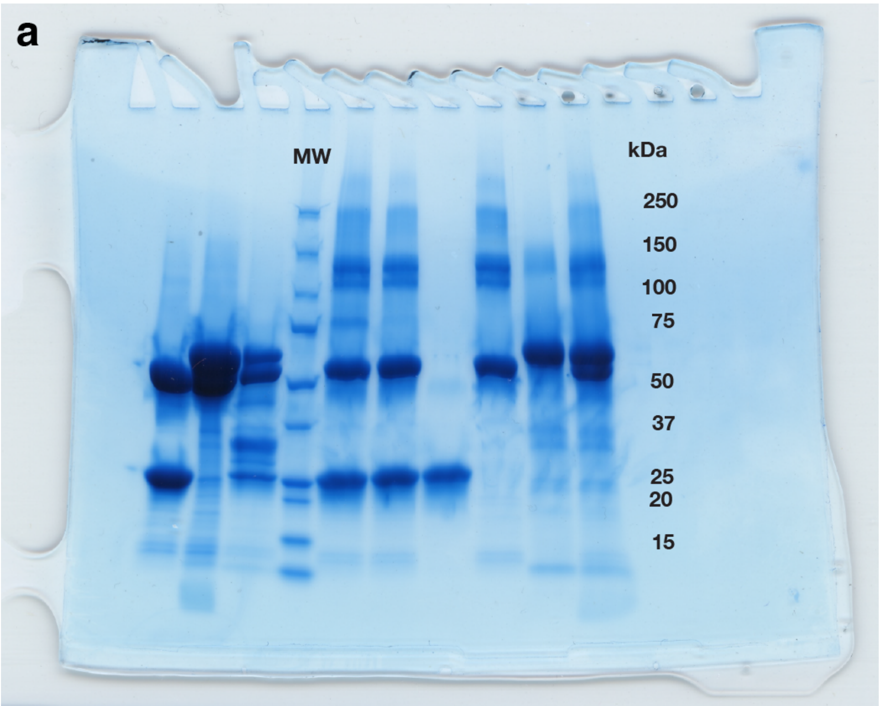

Supplementary Figure 8. (**a**) Uncropped gel of Supplementary Figure 4c.

Supplementary Tables

Supplementary Table 1. Phosphorylation and mutation sides of SPX domains localized between helix α6 and the adjacent domain.

**Protein**	**Organism**	
**Phosphorylation sites**[a]		
Vtc2	*S. cerevisiae*	S182, S187, S192, S193, S196
Vtc3	*S. cerevisiae*	S187, S190, S192, S195, S198
Gde1	*S. cerevisiae*	S254, T255, S256
Pho81	*S. cerevisiae*	T215
Pho91	*S. cerevisiae*	S295, S311, S312, T297
Syg1	*S. cerevisiae*	S342
SPX2	*A. thaliana*	S195
PHO1;H3	*A. thaliana*	S188
**Mutation site**[b]		
Xpr1	Human	L218S

[a] Reported in yeast database *thebiogrid.org* 4.2 and Arabidopsis database *PhosPhAt 4.0* [1–3].

[b] Reported in [4].

Supplementary Table 2. UniRef100 sequences, in which the helix α7 motif was identified.

Domain adjacent to SPX	UniProt identifier	Annotation	Species
TTM	A0A1G4IN84	---	*Lachancea sp.*
	A0A376B1S5	VTC3-like	*Saccharomycodes ludwigii*
	C8Z7T1	Vtc2p	*Saccharomyces cerevisiae*
	A0A1G4IPK6	---	*Lachancea dasiensis*
	J8Q8H7	Vtc2p	*Saccharomyces arboricola*
	A0A4T0X0U9	---	*Candida inconspicua*
	A0A0N7MMG7	---	*Lachancea quebecensis*
	A0A1X7QYL6	VTC2-like	*Kazachstania saulgeensis*
	A0A1L0CVH1	VTC3-like	*Hanseniaspora guilliermondii*
	G8JWC8	---	*Eremothecium cymbalariae*
	G0WEA1	---	*Saccharomyces dairenensis*
	J6EEI2	VTC3-like	*Saccharomyces kudriavzevii*
	A0A367Y0Z6	VTC2-like	*Candida viswanathii*
	Q750H3	---	*Eremothecium gossypii*
	A0A367YD17	VTC2-like	*Candida viswanathii*
	A0A1G4IWE9	---	*Lachancea nothofagi*
	N1P1U8	Vtc3p	*Saccharomyces cerevisiae*
	A0A1Q3AGE2	---	*Candida mogii*
	A0A1G4IN66	---	*Lachancea meyersii*
	A0A6C1E774	Pi metabolism transcription	*Saccharomyces pastorianus*
	A0A6C1EG68	VTC2 / 3	*Saccharomyces pastorianus*
	C5DXC4	---	*Candida mogii*
	A0A1G4M808	---	*Lachancea fermentati*
	A0A1Q3A2L8	---	*Candida mogii*
	J7R8F3	---	*Saccharomyces naganishii*
	A0A1S7HQC7	VTC2 / 3	*Zygosaccharomyces parabailii*
	G8ZR46	---	*Candida colliculosa*
	A0A1S7I1A1	VTC2 / 3	*Zygosaccharomyces parabailii*
	A0A1E3NSJ7	---	*Pichia membranifaciens*
	A0A6C1E346	VTC2 / 3	*Saccharomyces pastorianus*
	J8THM2	VTC2-like	*Saccharomyces kudriavzevii*
	A0A1B7TB83	---	*Hanseniaspora valbyensis*
	A0A0C7N0A4	---	*Lachancea lanzarotensis*
	Q02725	VTC3-like	*Saccharomyces cerevisiae*
	P43585	VTC2-like	*Saccharomyces cerevisiae*
	A0A0L8RA68	VTC3-like	*Saccharomyces eubayanus*
	A0A0L8RJY6	VTC2-like	*Saccharomyces eubayanus*
	A0A1E5R649	VTC2-like	*Hanseniaspora opuntiae*
	I2H317	---	*Tetrapisispora blattae*
	R9XJU5	---	*Ashbya aceri*
	B5VTE3	---	*Saccharomyces cerevisiae*
	A0A1Q2YBK4	---	*Pichia membranifaciens*
	A0A1B7SCC9	---	*Ogataea polymorpha*

	A0A0W0D5B2	VTC2-like	*Candida glabrata*
	C5DBH3	---	*Lachancea thermotolerans*
	A0A4C2E511	P_i metabolism transcription	*Zygosaccharomyces mellis*
	A0A0A8LCC9	---	*Kluyveromyces dobzhanskii*
	Q6CNJ4	---	*Kluyveromyces lactis*
	A0A1E5RES9	VTC2-like	*Hanseniaspora osmophila*
	W0T7N7	VTC2-like	*Kluyveromyces marxianus*
	C8ZJ09	Vtc3p	*Saccharomyces cerevisiae*
	Q6FUM8	---	*Candida glabrata*
	A0A1E5RD13	VTC2-like	*Hanseniaspora uvarum*
	A0A1G4K314	---	*Lachancea mirantina*
	A7TJR7	---	*Vanderwaltozyma polyspora*
	A0A0L8VRX1	VTC2p	*Saccharomyces boulardii*
	A0A0L8VFX1	VTC3p	*Saccharomyces boulardii*
	H2ASH6	---	*Kazachstania africana*
	B5VI22	---	*Saccharomyces cerevisiae*
	G0VD31	---	*Naumovozyma castellii*
ANK	A0A1E4RMD6	---	*Hyphopichia burtonii*
	A0A1L0FMB3	Glycerophosphocholine phosphodiesterase	*Hanseniaspora guilliermondii*
	A0A178FA43	Glycerophosphocholine phosphodiesterase	*Trichophyton rubrum*
	A0A022WCM4	---	*Trichophyton rubrum*
	D4DGV3	Glycerophosphocholine phosphodiesterase	*Trichophyton verrucosum*
	A0A1B7TH22	Glycerophosphocholine phosphodiesterase	*Hanseniaspora valbyensis*
	A0A1E5RTU3	Glycerophosphocholine phosphodiesterase	*Hanseniaspora opuntiae*
	A0A178FIU7	Glycerophosphocholine phosphodiesterase	*Trichophyton violaceum*
	A0A022Y3L0	---	*Trichophyton soudanense*
	D4B066	Glycerophosphocholine phosphodiesterase	*Arthroderma benhamiae*
	A0A1E5RJK4	Glycerophosphocholine phosphodiesterase	*Hanseniaspora uvarum*
	A0A2J6SUV1	Glycerophosphocholine phosphodiesterase	*Hyaloscypha bicolor*
	W7I643	Glycerophosphocholine phosphodiesterase	*Drechslerella stenobrocha*
	E4V1Y0	Glycerophosphocholine phosphodiesterase	*Arthroderma gypseum*
	G9P286	Glycerophosphocholine phosphodiesterase	*Hypocrea atroviridis*
ZnF	A0A6G1HV83	---	*Trichodelitschia bisporula*
	A0A5C3LW70	---	*Crucibulum laeve*
EXS	A8BME6	EXS family protein	*Giardia intestinalis*
	E1F2R9	EXS family protein	*Giardia intestinalis*
	A0A103XSP9	EXS family protein	*Cynara cardunculus*
	V6TBZ6	EXS family protein	*Giardia intestinalis*
	A0A3B3BYR1	Xenotropic and polytropic retrovirus receptor 1	*Oryzias melastigma*

Supplementary Table 3. Thermal stability of SPX2 in presence of ligands.

**Ligand**	**T_m [°C]**[a]
No ligand	34.4 ± 0.1
IP_6	47.2 ± 0.2
5-IP_7	43.8 ± 0.0
1,5-IP_8	38.2 ± 0.1

[a] Melting temperature determined by nanoDSF (Prometheus).

Supplementary References

1. Heazlewood, J. L. *et al.* PhosPhAt: a database of phosphorylation sites in Arabidopsis thaliana and a plant-specific phosphorylation site predictor. *Nucleic Acids Res.* **36**, D1015-1021 (2008).
2. Durek, P. *et al.* PhosPhAt: the Arabidopsis thaliana phosphorylation site database. An update. *Nucleic Acids Res.* **38**, D828–D834 (2010).
3. Zulawski, M., Braginets, R. & Schulze, W. X. PhosPhAt goes kinases—searchable protein kinase target information in the plant phosphorylation site database PhosPhAt. *Nucleic Acids Res.* **41**, D1176–D1184 (2013).
4. Legati, A. *et al.* Mutations in XPR1 cause primary familial brain calcification associated with altered phosphate export. *Nat Genet.* **47**, 579–581 (2015).
