# Supplementary material for: Inositol pyrophosphates activate the vacuolar transport chaperone complex in yeast by disrupting a homotypic SPX domain interaction
Source: Nat Commun. 2023 May 8;14:2645. doi: 10.1038/s41467-023-38315-w (PMC10167327; doi:10.1038/s41467-023-38315-w)
Supplement: Supplementary file 2 — Reporting Summary [file 41467_2023_38315_MOESM2_ESM.pdf]

nature portfolio

Corresponding author(s): Sebastian Hiller

Last updated by author(s): Mar 22, 2023

Reporting Summary

Nature Portfolio wishes to improve the reproducibility of the work that we publish. This form provides structure for consistency and transparency in reporting. For further information on Nature Portfolio policies, see our Editorial Policies and the Editorial Policy Checklist.

Statistics

For all statistical analyses, confirm that the following items are present in the figure legend, table legend, main text, or Methods section.

n/a	Confirmed	
☐	☒	The exact sample size (*n*) for each experimental group/condition, given as a discrete number and unit of measurement
☐	☒	A statement on whether measurements were taken from distinct samples or whether the same sample was measured repeatedly
☒	☐	The statistical test(s) used AND whether they are one- or two-sided<br>*Only common tests should be described solely by name; describe more complex techniques in the Methods section.*
☒	☐	A description of all covariates tested
☒	☐	A description of any assumptions or corrections, such as tests of normality and adjustment for multiple comparisons
☐	☒	A full description of the statistical parameters including central tendency (e.g. means) or other basic estimates (e.g. regression coefficient) AND variation (e.g. standard deviation) or associated estimates of uncertainty (e.g. confidence intervals)
☒	☐	For null hypothesis testing, the test statistic (e.g. *F*, *t*, *r*) with confidence intervals, effect sizes, degrees of freedom and *P* value noted<br>*Give P values as exact values whenever suitable.*
☒	☐	For Bayesian analysis, information on the choice of priors and Markov chain Monte Carlo settings
☒	☐	For hierarchical and complex designs, identification of the appropriate level for tests and full reporting of outcomes
☒	☐	Estimates of effect sizes (e.g. Cohen's *d*, Pearson's *r*), indicating how they were calculated

Our web collection on statistics for biologists contains articles on many of the points above.

Software and code

Policy information about availability of computer code

Data collection	All commercial instrumentation used for collecting the presented data is stated with their versions in the Methods section of the manuscript. Solution NMR: Bruker ASCEND 700 MHz spectrometer with TOPSPIN 3.7. Protein stability: Prometheus NT.48, Nanotemper Technologies Microscale Thermophoresis: Monolith NT.115, Nanotemper Technologies. Fluorescence plate reader: SPECTRAmax GEMINI XS, Molecular Devices.
Data analysis	Data was analyzed using standard software (PRISM 8.4.3, MATLAB R2017a)

For manuscripts utilizing custom algorithms or software that are central to the research but not yet described in published literature, software must be made available to editors and reviewers. We strongly encourage code deposition in a community repository (e.g. GitHub). See the Nature Portfolio guidelines for submitting code & software for further information.

Data

Policy information about availability of data

All manuscripts must include a data availability statement. This statement should provide the following information, where applicable:

- Accession codes, unique identifiers, or web links for publicly available datasets
- A description of any restrictions on data availability
- For clinical datasets or third party data, please ensure that the statement adheres to our policy

The NMR chemical shift assignments of SPX2 have been deposited in the BMRB data base with accession code 51877 [http://dx.doi.org/10.13018/BMR51877]. The

experimental data that support the findings of this study is shown in the article and its supplementary materials. The raw data underlying all Figures and Supplementary Figures is provided as a Source Data file. Any additional information required to reanalyze the data reported in this paper will be shared by the corresponding author upon request. Explanation: This combination of Data Bank Deposition / Source Data Provided / Data upon request is the most efficient combination of data sharing in terms of curation efforts vs. access frequency. PDB structures were accessed at https://www.rcsb.org/: Apo SPX4 (PDB: 5IIG [http://doi.org/10.2210/pdb5IIG/pdb] and PDB: 5IIQ [http://doi.org/10.2210/pdb5IIQ/pdb]), holo SPX4 (PDB: 5IJP [http://doi.org/10.2210/pdb5IJP/pdb]), human Xpr1 (PDB: 5IJH [http://doi.org/10.2210/pdb5IJH/pdb]), Gde1 (PDB: 5IJJ [http://doi.org/10.2210/pdb5IJJ/pdb]). The data base UniRef100 was accessed at https://www.uniprot.org/, the sequences assessed are given in Supplementary Table 1.

Human research participants

Policy information about studies involving human research participants and Sex and Gender in Research.

Reporting on sex and gender	N/A
Population characteristics	N/A
Recruitment	N/A
Ethics oversight	N/A

Note that full information on the approval of the study protocol must also be provided in the manuscript.

Field-specific reporting

Please select the one below that is the best fit for your research. If you are not sure, read the appropriate sections before making your selection.

☒ Life sciences ☐ Behavioural & social sciences ☐ Ecological, evolutionary & environmental sciences

For a reference copy of the document with all sections, see nature.com/documents/nr-reporting-summary-flat.pdf

Life sciences study design

All studies must disclose on these points even when the disclosure is negative.

Sample size	No statistical method was used to determine sample sizes and no sample size calculation was performed. The standard in the research field is to sample n>=3 independent measurements. Consequently, at least three independent replicates were performed for each condition in biochemical experiments and biophysics measurements and NMR measurements were averaged over at least 4 independent transients (scans).
Data exclusions	No data was excluded from the analyses.
Replication	Reported results were consistently replicated across multiple experiments. All replicates generated similar results. All experiments were carried out at least in triplicate, and the number of independent replicates is specified in each Figure caption. The individual free induction decays in the NMR experiments were averaged over at least 4 independent transients (scans).
Randomization	Randomization was not necessary, because all experimental parameters were precisely controlled and the experiments were performed with standardized ingredients. Randomization is typically not used in this field.
Blinding	Investigators were not blinded. Blinding during data collection was not needed because all experimental conditions were precisely controlled. Blinding during the analysis was not needed because the data was recorded by instruments, the results were quantitative and not subject to individual judgment or interpretation. Blinding is typically not used in the field.

Reporting for specific materials, systems and methods

We require information from authors about some types of materials, experimental systems and methods used in many studies. Here, indicate whether each material, system or method listed is relevant to your study. If you are not sure if a list item applies to your research, read the appropriate section before selecting a response.

Materials & experimental systems

n/a	Involved in the study	
☐	☒	Antibodies
☒	☐	Eukaryotic cell lines
☒	☐	Palaeontology and archaeology
☒	☐	Animals and other organisms
☒	☐	Clinical data
☒	☐	Dual use research of concern

Methods

n/a	Involved in the study	
☒	☐	ChIP-seq
☒	☐	Flow cytometry
☒	☐	MRI-based neuroimaging

Antibodies

Antibodies used	Antibodies to Vtc2 were generated in rabbits by Eurogentec, using the peptide DSVKDGSNDKKARWD (amino acids 32 to 46 of Vtc2) for immunization. The crude serum was used for Western blotting at 1:150 dilution in PBS with 5% milk powder.<br>Monoclonal mouse antibodies to the FLAG tag were purchased from Sigma (F1804-5MG) and used for Western blotting at a dilution of 1:300.<br>Secondary antibodies for Western Blot detection:<br>IRDye 800CW Goat anti-Rabbit IgG (H+L) N° 926-32211 used at a dilution of 1:1000.<br>IRDye® 800CW Goat anti-Mouse IgG (H + L), N° 926-32210 used at a dilution of 1:1000.
Validation	Specificity of the Vtc2 antibodies was tested on purified, recombinant SPX domain from Vtc2 (Wild et al., 2016) and on isolated vacuoles from wildtype and vtc2 knockout cells.<br>The commercial antibodies were validated by the vendor.
